# A cell-specific computational framework reveals a pan-cancer hypoxia signature predicting overall survival and ICI response

**DOI:** 10.1016/j.jbc.2025.111068

**Published:** 2025-12-17

**Authors:** Caiyu Zhang, Yitong Jin, Yifangfei Yu, Yuling Chen, Jiayi Yang, jialu Zhang, Qianyi Lu, Han Jiang, Yue Sun, Yakun Zhang, Hui Zhi, Yue Gao, Peng Wang, Shangwei Ning

**Affiliations:** 1 College of Bioinformatics Science and Technology, Harbin Medical University, Harbin, Heilongjiang, China; 2 Department of General Surgery, The Second Affiliated Hospital of Harbin Medical University, Harbin, China

**Keywords:** hypoxia, malignant cell-specific, pan-cancer, prediction of ICI response, single-cell RNA sequencing, survival prognostication

## Abstract

Hypoxia forms an immunosuppression environment and is involved in tumor immune escape, which may be the potential culprit of resistance to anticancer therapies. Nevertheless, there is still a lack of research that explores the characteristics of hypoxia in pan-cancer at the single-cell level and assesses the application of hypoxia in immune checkpoint inhibitor (ICI) efficacy and clinical outcomes. We delineated cell-specific hypoxia levels and developed a computational framework to generate a pan-cancer tumor hypoxia-related transcriptomic signature (HYP.SIG) using 38 scRNA-seq datasets encompassing 362 patients and 893,464 cells across 19 cancer types. We defined computational indicators of hypoxia levels as HYP.SIG scores to characterize the hypoxia status across 33 cancer types and 29 normal tissues within 18,901 samples. HYP.SIG scores exhibited cancer type-specific associations with genetic instability, and were linked to oncogenic signaling, poor response to ICI therapy, and impaired survival in multiple cancer types. Moreover, we established a predictive model for immunotherapy response utilizing six machine learning algorithms and 9 ICI cohorts (904 patients, four cancer types). HYP.SIG achieved better predictive performances in comparison to other previously established signatures. Subsequently, we applied three machine learning-based feature selection algorithms to filter HYP.SIG survival-related signatures and developed a prognostic model for predicting overall survival, incorporating clinical disease stages. Eventually, we screened four candidate therapeutic targets (*LDHA*, *SERF2*, *SLC2A1*, *NOP53*) for patients with tumors using 17 CRISPR cohorts and 1078 CRISPR cell lines. Overall, our study provides new ideas for survival prognostication, prediction of ICI response, and clinical therapeutic target development from the perspective of hypoxia.

Currently, cancer has become a major public health issue worldwide with poor prognosis and complex treatment challenges (1). Accumulating evidence has been reported that hypoxia plays a crucial role in the pathological progression of the disease (2). Hypoxia mediates malignant phenotypic transformation in various cancer entities. For example, hypoxia induces sarcoma and breast, colorectal, hepatocellular, prostate and uterine cancers to activate the transcription of numerous genes that are involved in biological processes such as angiogenesis, glucose and lipid metabolism, epithelial-mesenchymal transition, extracellular matrix (ECM) remodeling, cell motility, cancer stem cell specification, immune evasion, thereby driving tumor growth and invasion (3, 4). Although the significant functions of hypoxia in cancer progression have been extensively studied, the accurate quantification of hypoxia levels remains an important challenge. Furthermore, most research has primarily concentrated on individual cancer types or animal models (5), and the precise mechanisms by which hypoxia affects tumorigenesis and immunosuppression across different cancer types have not been fully elucidated. Therefore, there is an urgent need for a comprehensive molecular-level understanding and appreciation of hypoxia.

Biomarker studies are critical for characterizing the molecular features of hypoxia and optimizing patient selection to counteract immunoresistance. Traditional biomarker studies mainly focused on the analysis of whole exome sequencing or RNA sequencing technologies, which only reflect the average genetic profile of large populations of cells and ignore the heterogeneity of diverse cells (6). Immune checkpoint inhibitor (ICI) biomarkers identified from these studies exhibited low reliability and limited predictive values. In contrast, technological advances in single-cell RNA-seq enable us to detect the transcriptomes of individual cells and dissect gene expression in an unbiased manner (7). However, few transcriptomic signatures screened at single-cell resolution have been forthcoming. Hypoxia has variable effects on different patients and cell populations, scRNA-seq therefore could help us to develop biomarkers with better performance to portray tumor hypoxia and enhance patient survival benefit.

ICI, as a powerful clinical strategy in cancer treatment, provides a promising therapeutic option for patients with cancer (8). While ICIs has transformed the treatment landscape for multiple tumors, several serious challenges remain (9). Hypoxia, as a typical physiological trait in almost all solid tumors, contributes to impaired immune cell function and increased therapy resistance, which is a major barrier to cancer immunotherapy (10, 11, 12). Additionally, hypoxic signaling interacts with other signaling molecules to induce changes in gene expression across various biological processes (13). This may promote malignant behavior of cancer cells such as ‘inducing angiogenesis’, ‘tumor-promoting inflammation’, ‘activating invasion and metastases’, and ‘epithelial-mesenchymal transition’, which ultimately affects the outcomes of patients (14, 15, 16). Nevertheless, pre-existing predictive biomarkers for ICI outcome ignore a complex interplay between the immune system and the hypoxic tumor microenvironment (17). Therefore, it is of great necessity to center on defining new response biomarkers in cellular hypoxia.

In this study, we delineated the hypoxia status of different cell types by collecting and integrating multiple hypoxia gene sets and 38 scRNA-seq cohorts. We proposed a malignant cellular hypoxia-based computational framework to generate a pan-cancer hypoxia-related transcriptomic signature (HYP.SIG) at the single-cell level. Thereafter we characterized the hypoxic landscape and hypoxia-associated genomic alterations through introducing a computational metrics of hypoxia levels known as the HYP.SIG score and demonstrated a strong association between HYP.SIG, patient prognosis and ICI outcomes in large-scale data. The predictive models for patient survival probability and immunotherapy efficacy were constructed *via* a comprehensive analysis of pan-cancer transcriptomic data (11,057 patients, 33 cancer types) and 9 ICI cohorts (904 patients, four cancer types). Significantly, HYP.SIG showed sufficient performance across multiple cancer types. Ultimately, we determined four candidate therapeutic targets based on CRISPR data to further investigate the therapeutic value of HYP.SIG. Knocking out these HYP.SIG genes may enhance anti-tumor immunity and improve cancer therapy resistance.

## Results

### Single-cell analysis reveals a malignant cellular hypoxia-related transcriptomic signature in pan-cancer

**Figure 1:**
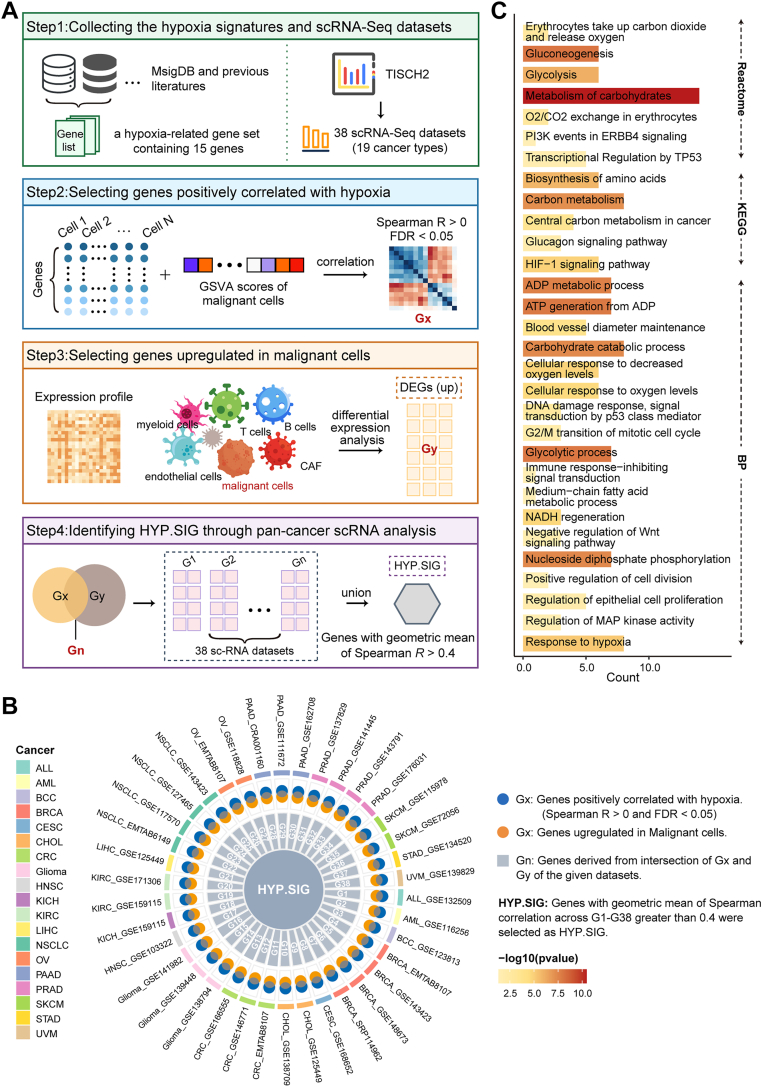
**Development and description of a malignant cell-specific hypoxia-related signatures through pan-cancer scRNA-seq analysis**. *A*, overview of the four-step computational framework for developing HYP.SIG. *B*, circus plot shows in detail the generation process of HYP.SIG. *C*, bar plot depicts the enriched Reactome, KEGG and BP pathways of the HYP.SIG. BP: biological process. HYP.SIG, hypoxia-related transcriptomic signature.

We suspected that hypoxia-associated transcriptomic alterations in tumor cells may represent an important predictor of poor clinical outcome and immunotherapy resistance of patients. With an established hypoxia-related gene set (18) (Ye_HYPOXIA), we delineated cellular hypoxia levels in five scRNA-seq datasets including cervical cancer, colorectal cancer, head and neck cancer, kidney cancer, and liver cancer. We performed gene set variation analysis (GSVA) analysis of all cells in these gene sets based on Ye_HYPOXIA, the result revealed that tumor cells showed higher GSVA scores compared to other cell populations such as endothelial cells, epithelial cells, fibroblasts, CD8T cells, B cells (Fig. S1). Accordingly, we employed a four-step computational framework to develop HYP.SIG which could reflect the hypoxia level of the tumor (Fig. 1*A*). We performed Spearman correlation analysis between gene expression levels and GSVA scores (based on HYP.SIG) of malignant cells among 38 scRNA-seq datasets included 19 cancer types. Genes that exhibited a positive correlation with GSVA scores were regarded as ‘Gx’ (Spearman cor > 0, FDR < 0.05). Genes differentially up-regulated in malignant cells were regarded as ‘Gy’ (logFC ≥ 0.25 and FDR < 0.05). ‘Gx’ and ‘Gy’ were intersected in each dataset to yield ‘Gn’, which represented tumor-specific genes positively associated with hypoxia (Fig. 1*B*). Subsequently, the geometric mean Spearman correlation was calculated for each gene among G1-G38 gene sets. Ultimately, genes with geometric mean Spearman correlation higher than 0.4 were selected as HYP.SIG, which contained 68 genes.

To investigate the biological functions of the 68 genes in HYP.SIG, we performed functional enrichment analysis with BP data from GO terms, the KEGG database, and the reactome pathway database. Here, we found that HYP.SIG were primarily enriched in pathways related to hypoxia, energy metabolism, DNA damage, cell growth and angiogenesis, such as HIF-1 signaling pathway, glycolysis, ATP generation, cell cycle, PI3K signaling, mitogen-activated protein kinase signaling and WNT signaling. In addition, HYP.SIG was enriched in pathways correlated with immune, including immune response-inhibiting signal transduction (Fig. 1*C*).

### A pan-cancer integrative evaluation implied that hypoxia status is present in a wide range of solid tumors

**Figure 2:**
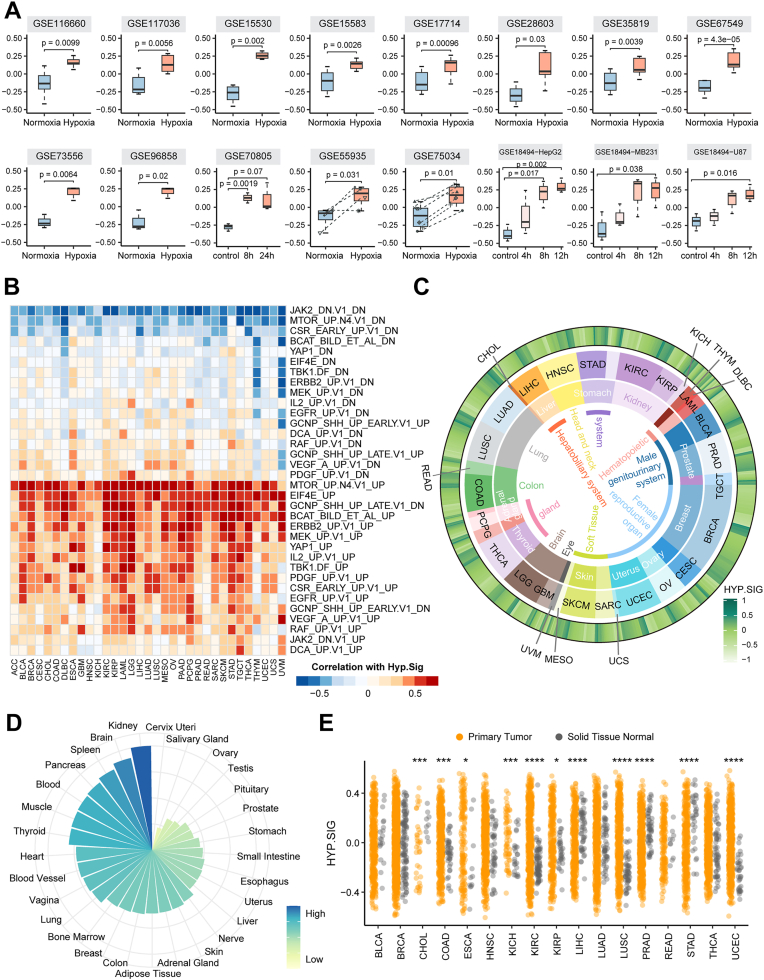
**Integrative measurement of hypoxia levels in tumor and normal tissues**. *A*, box plots show the HYP.SIG scores for hypoxia and normoxia samples across 14 datasets. The *t* test *p*-values are stated. *B*, heatmap shows the relationship between the HYP.SIG score and GSVA scores of C6 oncogenic processes in 33 TCGA cancer types. *C*, the circle heatmap depicts the average HYP.SIG scores per cancer type. Tissue types, cancer types and average HYP.SIG scores are shown from the inner circle to the outer circle. *D*, average HYP.SIG scores across normal tissues in the genotype-Tissue Expression dataset. *E*, *dot plo*t shows higher HYP.SIG scores in primary tumors (*orange*) compared to adjacent normal tissues (*gray*) among several cancer types. The statistical difference was analyzed by a paired *t* test, where ∗∗∗∗ represents *p* < 0.0001. ∗∗∗ represents *p* < 0.001, ∗∗ represents *p* < 0.01. ∗ represents *p* < 0.05. HYP.SIG, hypoxia-related transcriptomic signature; GSVA, gene set variation analysis; TCGA, The Cancer Genome Atlas.

We demonstrated the characteristics of HYP.SIG in detail and evaluated the reliability of HYP.SIG in representing hypoxia levels. First, we compared the expression patterns of HYP.SIG across 18 cancer types with at least five matched tumor and normal samples (Fig. S2*A*). Almost all HYP.SIG exhibited remarkable differential expression. Moreover, most of the HYP.SIG genes were significantly higher expressed in cancer samples compared to normal samples. Next, we tested whether HYP.SIG could be represented to assess the hypoxia level. We collected 34 independent datasets containing hypoxic and normoxic samples across multiple cancer types (Table S8), and calculated the areas under the curve (AUCs) for each HYP.SIG gene distinguishing between known hypoxic and normoxic samples based on expression values. We found that the majority of genes in HYP.SIG had AUC values higher than 0.7, except for *NOP53*, which lacked expression profiles in all 33 datasets (Fig. S2*B*). Moreover, in most cases, cells under the hypoxia conditions exhibited significantly higher hypoxia scores than those under the normoxia conditions (Fig. 2*A*). These results presented that the pan-cancer gene signature we generated could specifically reflect the properties of hypoxic malignant cells. We then calculated a hypoxia score for each sample in The Cancer Genome Atlas (TCGA) dataset based on HYP.SIG. The scores for HYP.SIG were positively associated with hypoxia scores based on other established hypoxia signatures, especially Hallmark_Hypoxia, a gold standard for characterizing tumor hypoxia (Fig. S3, *A* and *B*). The observed consistency suggested that HYP.SIG was reliable. We also compared AUC values for scores based on various hypoxia signatures distinguishing known hypoxic and normoxic samples. It was found that HYP.SIG performed well compared to other previously published signatures, exhibiting average AUC higher than those of Buffa and Winter, which are widely used hypoxia signatures, and similar to that of Hallmark_Hypoxia (Fig. S3*C*). Finally, we observed HYP.SIG scores were correlated with oncogenic signatures related to energy metabolism and cell growth (Fig. 2*B*). Our results were in agreement with previous notions that hypoxia leads to metabolism alterations, regulates cell proliferation, and induces inflammatory responses. Briefly, we successfully developed a tumor-specific pan-cancer signature to assess the level of hypoxia, which can further portray the biological characteristics of HYP.SIG in different cancers.

Subsequently, we calculated transcriptomic signature scores for TCGA cancer patients by applying the GSVA algorithm to HYP.SIG. It could systematically delineate hypoxia landscape among 33 cancer entities, revealing the specificity of hypoxia levels across cancer types. In general, solid tumors originating from central nervous system, urinary system, reproductive system, digestive system, chest, head and neck tissues (glioblastoma multiforme (GBM), low grade glioma (LGG), kidney renal clear cell carcinoma (KIRC), bladder urothelial carcinoma (BLCA), cervical squamous cell carcinoma and endocervical adenocarcinoma (CESC), pancreatic adenocarcinoma (PAAD), lung adenocarcinoma (LUAD), lung squamous cell carcinoma (LUSC), head and neck squamous cell carcinoma (HNSC)) exhibit higher HYP.SIG scores (Fig. 2*C*). VHL inactivation is a common event in KIRC, leading to constitutively high levels of hypoxia-inducible factor (HIF) (19). Consistently, KIRC exhibited relatively higher HYP.SIG scores compared to most cancer types (Fig. S4*A*). A similar trend was observed for the Hallmark_Hypoxia gene set (Fig. S4*B*). Moreover, HYP.SIG was significantly positively correlated with HIF target genes, including *VEGFA*, *CA9*, *NDRG1*, *EGLN3*, and *SLC2A1*, in KIRC (Fig. S4*C*). These results collectively supported the validity of HYP.SIG. We then assessed hypoxia levels in more than 9000 samples from the genotype-tissue expression database using the same algorithm and observed similar distributions as in the TCGA database (Fig. 2*D*). The tendency of hypoxia distribution suggested that hypoxic degrees of cancers may be tissue type specific. Moreover, most primary tumors had higher HYP.SIG scores and greater variations compared to adjacent normal tissues, particularly in KIRC, LUSC, and uterine corpus endometrial carcinoma (UCEC), revealing the heterogeneity of hypoxia levels in both tumor and normal tissues at the pan-cancer level (Fig. 2*E*). Our findings elucidated that hypoxia is an ideal environment for cancer cell growth and prolonged hypoxia plays a contributory role during tumor progression. Given the above, we developed a pan-cancer transcriptomic signature, termed HYP.SIG, which could reliably quantify hypoxia levels and systematically depict the hypoxia landscape across 33 cancer entities and 29 tissues.

### Tumor hypoxia is associated with cancer type-specific genomic variations

**Figure 3:**
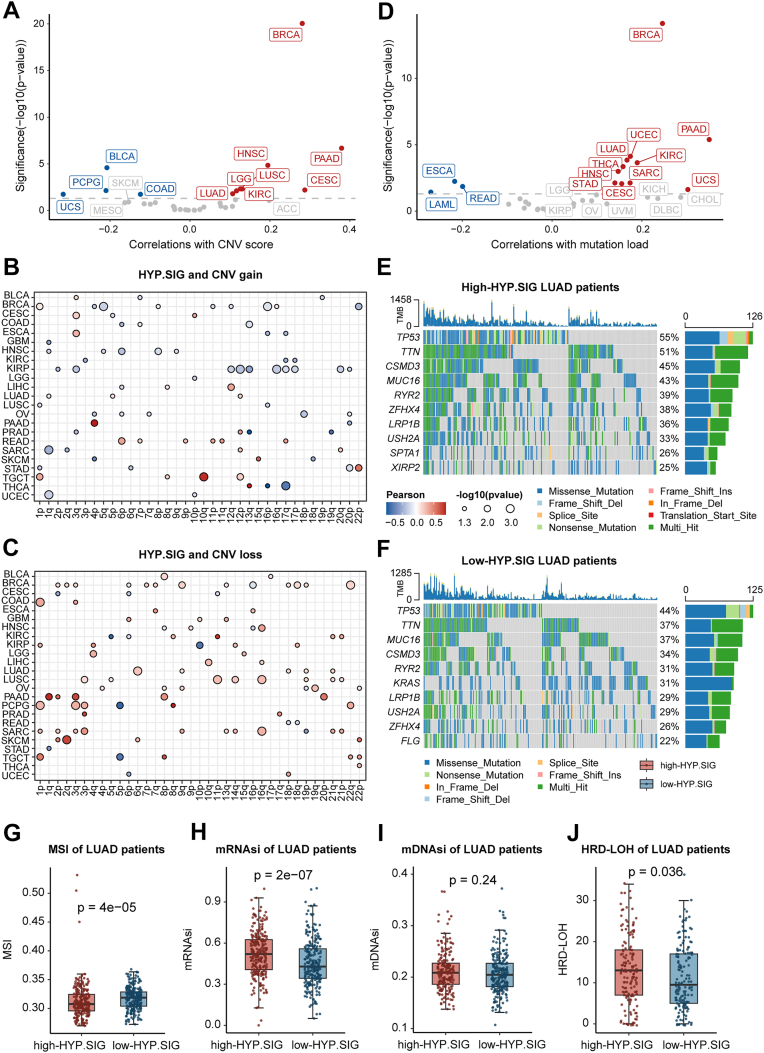
**Associations between hypoxia levels and genomic variations at the pan-cancer level**. *A*, correlations between HYP.SIG scores and copy number variation (CNV) scores in TCGA cancer types, with correlation coefficients and significance displayed on the x-axis and y-axis. Cancers with a significant positive and negative association between HYP.SIG score and CNV score are marked in *red* and *blue*, respectively. Non-significant cancers are showed in *grey*. The *grey* line indicates a *p*-value of 0.05. *B* and *C*, dot plots show correlations between CNV scores and arm-level copy-number gains and copy-number losses. *D*, correlations between HYP.SIG scores and mutation loads in TCGA cancer types. *E* and *F*, waterfall plots show the top mutation events for each LUAD patient in the high- and low-HYP.SIG groups, respectively. Bar plots in the *top panel* illustrate the HYP.SIG scores per patient. Statistical graph of mutation events for each gene is shown in the *right panel*. Colors are variant classifications. *G* and *J*, comparisons of microsatellite instability (*G*), stemness indices *H* and *I*, and LOH scores (*J*) for LUAD patients in the high-HYP.SIG and low-HYP.SIG cohorts. The statistical difference was analyzed by the Wilcoxon test. HYP.SIG, hypoxia-related transcriptomic signature; LUAD, lung adenocarcinoma; TCGA, The Cancer Genome Atlas.

Genomic instability is known to be an important hallmark of cancer (20). The development of cancer undergoes a complex succession of genetic alterations that confer a selective growth advantage on cells gradually transforming into cancer cells (21). Hypoxia, however, is one of the key factors in the tumor microenvironment and can promote genetic instability (22). Therefore, to elucidate how genomic alterations vary with hypoxia in different cancers, we investigated the relationship between hypoxia levels and somatic copy number variants (SCNAs) and single nucleotide variants (SNVs). For SCNAs, we first calculated the SCNA score for each sample, which was defined as the sum of focal-, arm-, and chromosome-level SCNA scores. By calculating the Spearman correlation, we found significant associations between the HYP.SIG score and SCNA score in 12 cancer types (Fig. 3*A*). Cancer types with higher levels of hypoxia showed a significant strong positive correlation, such as CESC, HNSC, KIRC, LGG, LUAD, LUSC. Next, we evaluated the relationship between the HYP.SIG score and arm-level SCNA gains and losses across cancer types. Arm-level gains were more remarkably correlated with the HYP.SIG score than arm-level losses. Meanwhile, arm-level SCNA gains predominantly showed positive correlations with the HYP.SIG score, while the arm-level losses did the opposite (Fig. 3, *B* and *C*). These results suggested that genomic instability increases with the degree of tumor hypoxia in specific cancer types.

For SNVs, 14 cancer types exhibited a notable relationship between the HYP.SIG score and mutation load by calculating the Spearman correlation, and cancer types with higher levels of hypoxia were also showed a positive correlation with mutational load (Fig. 3*D*). In general, the HYP.SIG score for several cancer types with a higher level of hypoxia was associated with both SCNA and SNV in cancer type-specific analyses (*e*.*g*.*,* CESC, HNSC, KIRC, and LUAD). Besides that, tumor hypoxia was first discovered in lung cancer, we further selected LUAD to examine the discrepancy in mutational events between the high- and low-HYP.SIG groups (according to the median HYP.SIG score). Specifically, we found that overall mutation events were mainly contributed by missense mutations (Fig. 3, *E* and *F*). We also observed the top mutated oncogenes in individual groups and found that *TP53*, *TNN*, *CSMD3* and *MUC16* were more frequently mutated in the high-HYP.SIG LUAD patients, which was confirmed by previous studies. Variations in the cell cycle-related gene *TP53* are one of the most common somatic events in cancer, *TP53* mutations promote tumor formation and affect resistance to immunotherapy of patients (23). Meanwhile, co-mutations in *TP53* and *TTN* also inhibit multiple immune-related signaling pathways (24). *CSMD3* and *MUC16* mutations are highly correlated with increased tumor mutation burden (TMB) and poor clinical prognosis (25, 26). These above results revealed that patients with high-HYP.SIG score may exhibit more malignant characteristics. Microsatellite instability (MSI) is a unique molecular alteration in hypermutated tumors, caused by defects in DNA mismatch repair (27). The low-HYP.SIG group of LUAD patients presented the MSI-high scores, implying that high mutational loads in lung cancer patients may not be due to the presence of MSI-high tumors (Fig. 3*G*). To better understand genomic instability, we investigated the association of HYP.SIG with DNA repair deficiency and tumor stemness indices in lung cancer (Fig. 3, *H*–*J*). Elevated mRNAsi, mDNAsi and HRD scores were displayed in the high HYP.SIG subgroup, and we found a prominent gap in both mRNAsi and HRD analyses between these two groups (Wilcoxon test, *p* < 0.05). This may shed light on other possible carcinogenic mechanisms.

Taken together, positive correlations of HYP.SIG scores with SCNAs and SNVs manifest that cancer cells with an increased level of hypoxia tend to harbor a more unstable genome than normoxic cells in several cancer types.

### HYP.SIG serves as a pan-cancer prognostication on signaling pathways, ICI treatment response, and clinical outcomes

**Figure 4:**
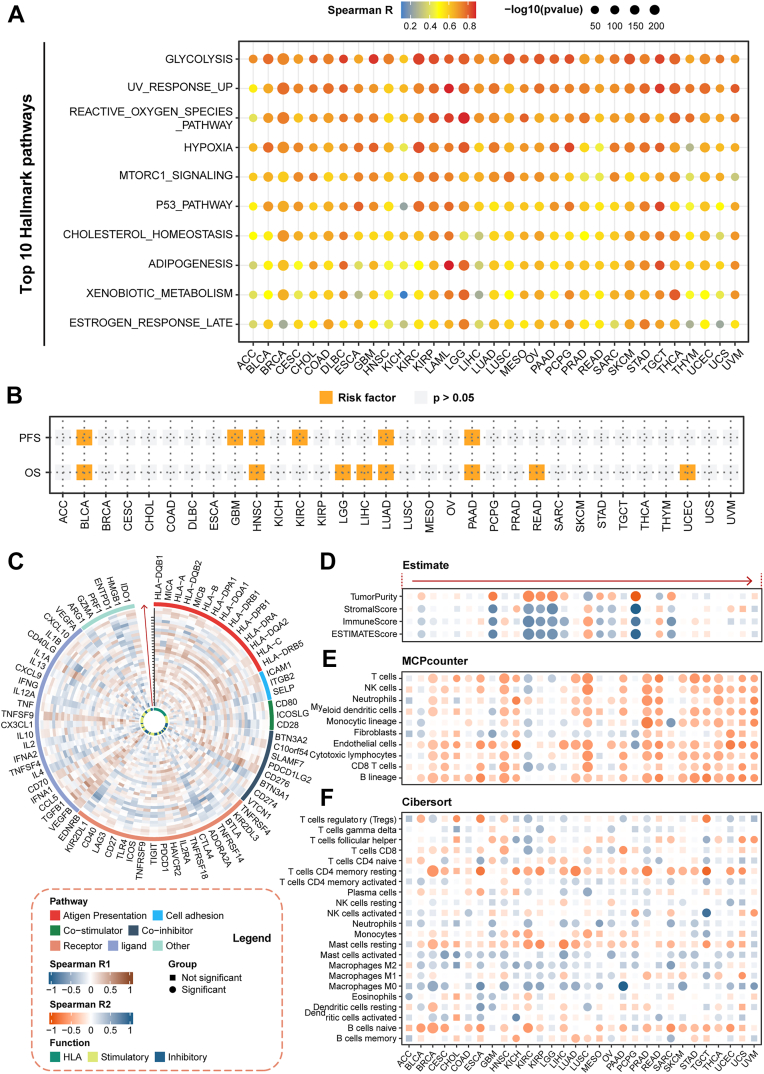
**Pan-cancer analysis of HYP.SIG**. *A*, heatmap depicts the correlation between HYP.SIG scores and the Top 10 hallmark pathways across 33 TCGA cancer types. *B*, the relationship between HYP.SIG scores and OS/PFS of patients in TCGA pan-cancer cohorts. The *p* value < 0.05 and HR > 1 was considered as a risk factor. *C*, *circle plot* depicts the association between HYP.SIG and the expression level of immune checkpoints for individual cancer types. The *black arrow* on the vertical axis represents different cancer types, corresponding to the annotations on the x axis in plot D. *D*, spearman correlations between HYP.SIG scores and the ESTIMATE score, immune score, and stromal score by ESTIMATE algorithm. *E*, spearman correlations between HYP.SIG scores and the infiltrating immune cells estimated through Microenvironment Cell Populations-counter. *F*, spearman correlations between HYP.SIG scores and the absolute abundance of 22 immune cell types estimated through CIBERSORT. HYP.SIG, hypoxia-related transcriptomic signature; TCGA, The Cancer Genome Atlas.

First of all, we examined the hallmark pathways concerning the expression of HYP.SIG. Malignant tumors with high HYP.SIG exhibited enrichment in metabolism pathways, TP53 pathways, ROS, andmammalian target of rapamycin (MTOR) signaling (Fig. 4*A*). All of these pathways facilitated cancer cell survival, proliferation and metabolism in accordance with previous studies. Afterwards, we evaluated the connection between the HYP.SIG score and clinical outcomes of patients in the TCGA cohort. High HYP.SIG scores were markedly related to impair overall survival across eight cancer types, including BLCA, HNSC, LGG, liver hepatocellular carcinoma (LIHC), LUAD, PAAD, rectum adenocarcinoma (READ), and UCEC. In addition, high HYP.SIG scores were correlated with poor progression free survival across six other cancers, including BLCA, GBM, HNSC, KIRC, LUAD, and PAAD (Fig. 4*B*).

Immune cells dominate the major cellular compartment of tumor lesions. Tumor immunity can stimulate tumor growth and affect patient prognosis. In the end, we investigated the impact of hypoxia on tumor immunity thoroughly. A correlation analysis of the HYP.SIG score and the expression of immune checkpoint genes was conducted (Table S9). Hypoxia levels were generally positively correlated with these checkpoint genes, such as CD274 and CTLA4, in various kinds of cancers (Fig. 4*C*). This finding is consistent with previous reports showing that hypoxia *via* HIF-1α up-regulates PD-L1 expression in certain tumors (28, 29). Furthermore, increased expression levels of CTLA4 and PDCD1 were also observed in tumor patients exhibiting hypoxia (30). Thereafter, the ESTIMATE algorithm was applied to assess the relationship between the HYP.SIG score and immune characteristics at the pan-cancer level. We identified a considerably positive correlation between HYP.SIG scores and ESTIMATE scores, stromal scores, and immune scores by calculating the Spearman correlation. Conversely, tumor purity exhibited a negative correlation (Fig. 4*D*). In the next part, we evaluated the infiltrating immune cells in individual cancer types. Cytotoxic immune cells, which are vital factors of the immune system against cancer, were found decreased in tumors with high HYP.SIG by the Microenvironment Cell Populations-counter (MCP-counter) method, including CD8+T cells, NK cells and macrophages (Fig. 4*E*). Moreover, we implemented CIBERSORT (31) to deconvolve transcriptomes and calculate the proportions of 22 immune cells in tumor samples. Antitumor-related immune cells, including M1 macrophages, CD8+T cells, and memory CD4 T cells, demonstrated a negative association with the HYP.SIG score in nearly all cancers, whereas tumor-promoting immune cells, such as M2 macrophages, showed a positive association (Fig. 4*F*). These results were remarkable resemble, indicating that hypoxia may inhibit anti-tumor immunity. On the whole, we successful generated a tumor-specific, hypoxia signature that acts as a pan-cancer prognosticator of oncogenic signaling, poor tumor response to ICI, and impaired patient survival. This could boost constructing models for predicting immunotherapy response and clinical outcomes of patients.

### HYP.SIG-based immunotherapy outcome predictive model exhibits sufficient performance

Considering the dramatic association between the hypoxia level and the tumor immunity, we intended to develop a pan-cancer prediction model based on HYP.XIG to explore its potential value in predicting immunotherapy response. First, we portrayed HYP.SIG-related immune molecular characteristics. We recognized pivotal interaction pairs of immune checkpoints associated with HYP.SIG, such as PD1 and its ligand PD-L1. The high HYP.SIG subgroup maintained higher expression of PD-L1 transcripts in the majority of cancers (Fig. S5*A*). TMB serves as a promising pan-cancer predictor of ICI response, with higher levels indicating better immune response, whereas the opposite is true for HYP.SIG. Interestingly, a seemingly controversial result delineated that TMB was positively correlated with the HYP.SIG score across cancer types (Fig. S5*B*). To thoroughly understand the relevance of antitumor immunity with HYP.SIG and TMB, we divided patients into four subgroups based on the median HYP.SIG score and median TMB: high HYP.SIG/high TMB (HPHT, indicating high HYP.SIG and high TMB), high HYP.SIG/low TMB (HPLT, indicating high HYP.SIG and low TMB), low HYP.SIG/high TMB (LPHT, indicating low HYP.SIG and high TMB), and low HYP.SIG/low TMB (LPLT, indicating low HYP.SIG and low TMB). We then compared the infiltration levels of immune cells across four subgroups. Higher levels of immune cell infiltration emerged in the low HYP.SIG subgroup compared to the high HYP.SIG subgroup by the MCP counter. Similarly, half of immune cell types in the low TMB subgroup also exhibited higher infiltration. High tumor hypoxia could suppress the killing function of immune cells, promoting tumor immune escape. Low TMB level leads to a lack of immunogenic neoantigens generated by tumor mutations, which results in decreased anti-tumor immunity. As anticipated, the infiltration of cytotoxic lymphocytes decreased in both HP and LT subgroups. Moreover, the comparison of immune cell infiltration among the four subgroups demonstrated that tumors with low HYP.SIG displayed significantly better anti-tumor immunity regardless of TMB level (Fig. S6, *A* and *B*).

**Figure 5:**
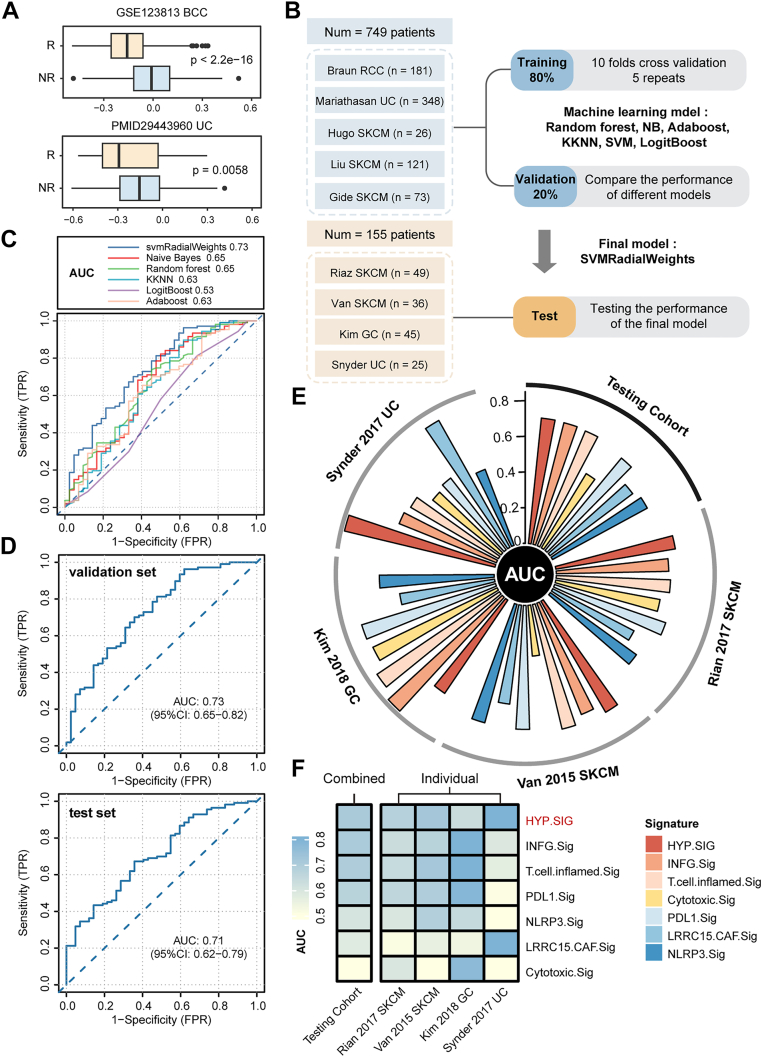
**Development and evaluation of a predictive model for ICI outcomes**. *A*, differences in HYP.SIG scores between responders and nonresponders of immunotherapy (top: basal cell carcinoma (BCC) patients; bottom: urothelial carcinoma (UC) patients). *B*, the workflow of training, validating, and testing the HYP.SIG-related predictive model developed by machine learning algorithms. *C*, the ROC curve illustrates the predictive performance of different algorithms in the validation set. *D*, ROC plot presents the performance of the final HYP.SIG-related predictive model in the validation and testing sets. *E*, circus and (*F*) heatmap plots depict the comparison between the predictive value of HYP.SIG and previously published pan-cancer signatures among different testing sets. HYP.SIG, hypoxia-related transcriptomic signature; ROC, receiver operating characteristic.

Subsequently, we explored the predictive potential of HYP.SIG on tumor ICI therapy at the bulk level and scRNA-seq level, respectively. The results from both cohorts revealed higher HYP.SIG scores in immunotherapy non-responders compared to responders (Fig. 5*A*). Collectively, our findings manifest compelling evidence for a connection between hypoxia levels and immunotherapy outcomes in patients, providing the possibility for further constructing models to predict ICI efficacy. We employed bulk-level transcriptome data and clinical information from six ICI cohorts, with the workflow illustrated in Figure 5*B*. We trained models using six machine learning algorithms and optimized their parameters through five repetitions of ten-fold cross-validation. Subsequently, the AUC was estimated for each model and compared in the validation cohort, with AUC values ranging from 0.53 (LogitBoost) to 0.73 (svmRadialWeights) (Fig. 5*C*). We selected the “support vector machine” machine learning algorithm model with the highest AUC as the final HYP.SIG model and further tested the prediction performance of this model in the independent testing cohort with an AUC of 0.71 (Figs. 5*D*, S7). We evaluated the predictive performance of HYP.SIG by comparing it with established pan-cancer models for ICI response prediction, including INFG.Sig, T.cell.inflamed.Sig, PDL1.Sig, NLRP3.Sig, LRRC15.CAF.Sig, and Cytotoxic.Sig (Fig. 5, *E* and *F*). As shown, HYP.SIG model delivered best performance in the external testing set, with a slightly higher AUC. What's more, most pan-cancer signatures did not report ideal performance across all cohorts. Specifically, AUC values of INFG.Sig and T.cell.inflamed.Sig, which had high predictive accuracy in the testing set, were both lower than 0.6 in Snyder 2017 UC. Nevertheless, HYP.SIG-based model achieved consistently good effectiveness in all cohorts covering three cancer types, with the lowest AUC value of 0.63 in Kim 2018 GC. Collectively, our results confirmed the feasibility and reliability of the predictive mode based on HYP.SIG for assessing response to anti-PD-L1/PD1 or anti-CTLA-4 immunotherapy at the pan-cancer level.

### Constructing and validating a HYP.SIG-related predictive model for OS in pan-cancer

**Figure 6:**
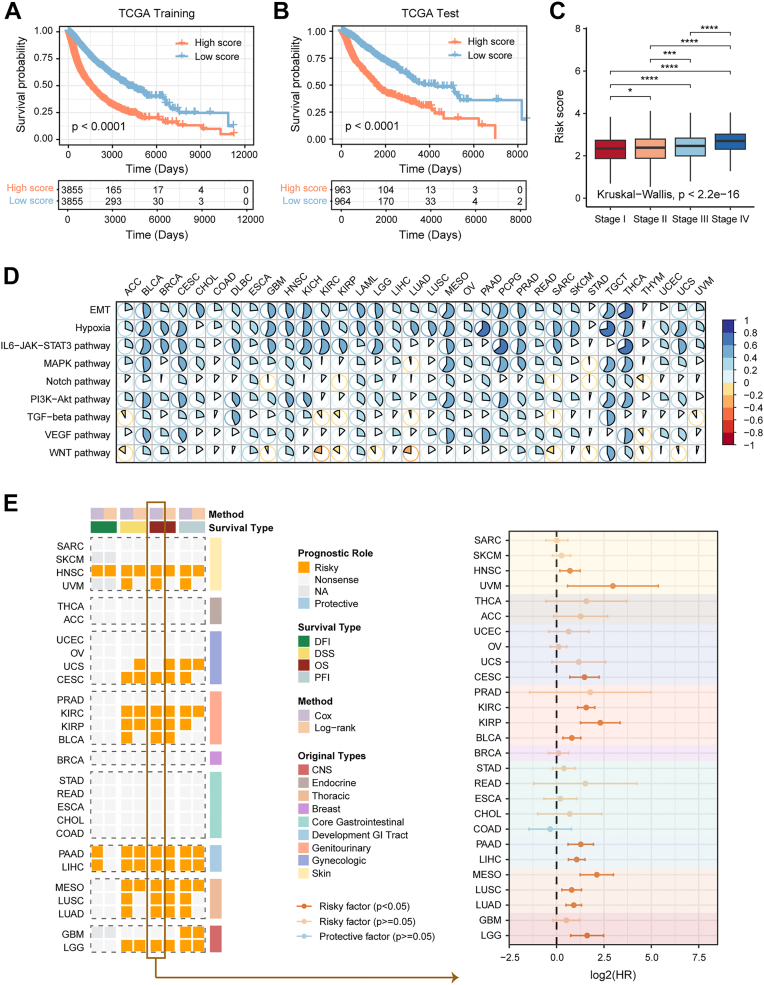
**Construction and evaluation of a predictive model for overall survival in TCGA**. *A* and *B*, Kaplan-Meier curves show the overall survival between high- and low-risk patients in TCGA pan-cancer training and testing sets. *C*, *box plot* shows the differences of HYP.SIG-related risk scores among various tumor stages in TCGA pan-cancer cohort. The statistical difference was analyzed by the Wilcoxon and Kruskal-Wallis test. *D*, heatmap shows the Spearman correlation of HYP.SIG-related risk score and enrichment of tumor-promoting pathways in the TCGA cohort. *E*, the correlation between risk scores and clinical outcomes (OS, DFI, disease-specific survival, and PFI) was evaluated through univariate Cox regression analysis and the log-rank test in the TCGA dataset. The *p* value < 0.05 and HR > 1 was regarded as a risk factor. The *p* value ≥ 0.05 was regarded as nonsense. ‘‘N/A’’ denotes unavailable data. The forest plot on the *right* shows the univariate Cox regression result of the risk score. HYP.SIG, hypoxia-related transcriptomic signature; TCGA, The Cancer Genome Atlas.

Given the remarkable associations between the HYP.SIG score and clinical outcomes in specific cancer types, particularly OS, we optimized HYP.SIG to develop a pan-cancer prognostic model. Initially, the 68 HYP.SIG genes were subjected to least absolute shrinkage and selection operator, random forest and boruta and extreme gradient boosting (XGBoost) in the pan-cancer TCGA cohort, respectively (Fig. S8, *A* and *B*). The 29 common genes of the three algorithms were utilized to develop the predictive model using a stepwise Cox regression analysis in the TCGA pan-cancer training set. Ultimately, the risk score was generated for each patient by integrating the expression values of 17 genes with their Cox coefficients (Fig. S8*C*). We divided patients in the TCGA training and test sets into high and low risk groups based on the median risk score. Patients with higher risk scores exhibited adverse survival outcomes in both cohorts (Fig. 6, *A* and *B*). Meanwhile, patients with higher clinical stage were associated with higher risk scores in the total TCGA pan-cancer cohort (Fig. 6*C*). Subsequently, we evaluated the relationship between HYP.SIG-related risk scores and tumor-promoting signaling pathways to investigate whether tumor-promoting biological functions were upregulated in patients with a high-risk score. HYP.SIG-related risk score was positively correlated with the GSVA score for most tumor-promoting pathways in virtually all TCGA cancer types (Fig. 6*D*). Moreover, we observed that the HYP.SIG-related risk score stressed superior predictive potential for clinical outcomes in various other cancer types, including BLCA, CESC, HNSC, KIRC, kidney renal papillary (KIRP), LGG, LIHC, LUAD, LUSC, mesothelioma (MESO), PAAD, UCS, and was a critical risk factor for OS (Figs. 6*E*, S9*A*). We further tested the robustness of this model by calculating a risk score of patients in several external validation cohorts using the same methodology (Fig. S9*B*). Notably, the HYP.SIG-related risk score also performed well among these datasets, indicating that our pan-cancer predictive model developed based on HYP.SIG exhibited stable and reliable performance under different conditions.

### Pan-cancer prognostic model combining clinical stage further strengthen the predictive power

**Figure 7:**
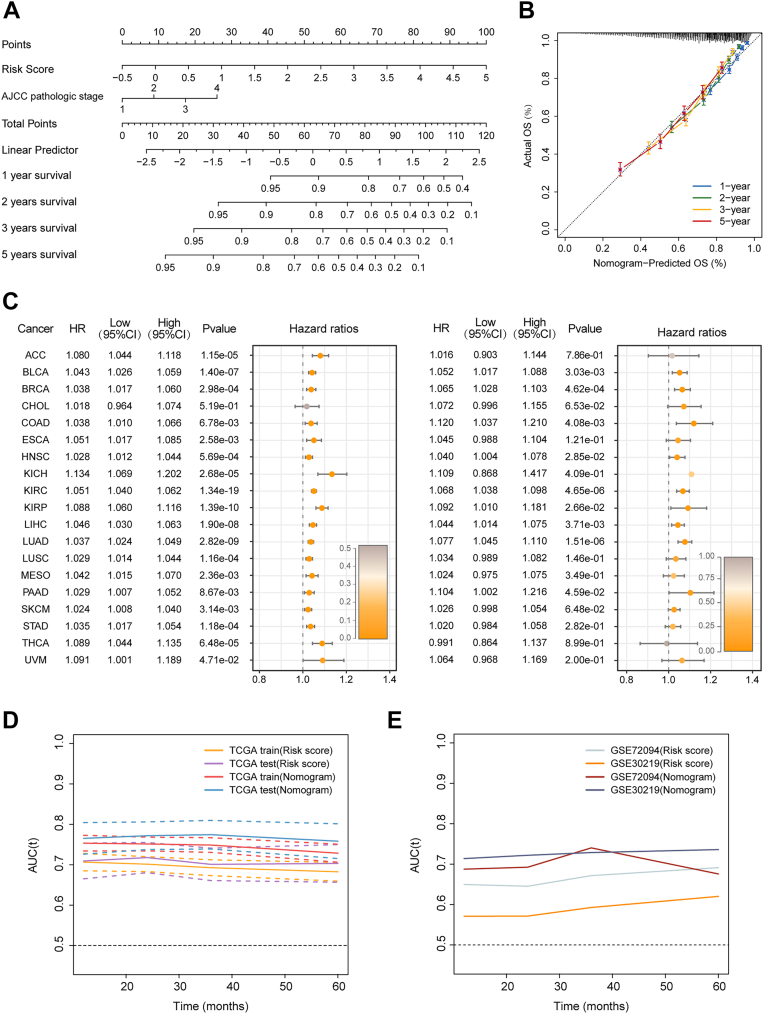
**Assessment of the HYP.SIG-related prognostic model combining clinical stage in pan-cancer**. *A*, prediction of overall survival for patients in the TCGA pan-cancer cohort using nomograms. *B*, the calibration curve shows the consistency between predictions and real survival. *C*, forest plots show the results of univariate Cox regression for the nomogram score within TCGA pan-cancer training and testing sets. *D* and *E*, time-dependent ROC analysis illustrates the predictive performance of the HYP.SIG-related risk score and nomogram score in TCGA pan-cancer training and testing sets (*D*) and external validation sets (*E*). HYP.SIG, hypoxia-related transcriptomic signature; ROC, receiver operating characteristic; TCGA, The Cancer Genome Atlas.

In order to better predict prognosis of patients across distinct cancer types and enhance the robustness and clinical value of the HYP.SIG-related risk score, we generated a nomogram score incorporating both clinical stage and risk score within the TCGA pan-cancer cohort (Fig. 7*A*). The calibration curves of disease-specific survival at 1, 2, 3, 5 years after cancer diagnosis revealed a high degree of concordance between predicted survival probability and actual survival, suggesting that the nomogram score was a feasible and reliable predictor for prognostication (Fig. 7*B*). We described the impact of the nomogram score on overall survival in the TCGA pan-cancer training and validation sets by univariate Cox analysis, demonstrating that the nomogram score was a risk factor for poor prognosis across most cancer types (Fig. 7*C*). Notably, time-dependent receiver operating characteristic (ROC) curves revealed that AUC values predicted by the nomogram score combined with pathologic stage performed better compared to the HYP.SIG-related risk score alone in both the TCGA pan-cancer training cohort and validation cohort (Figs. 7*D*, S10, *A* and *B*). Meanwhile, this nomogram score also showed relatively good predictive potential in external validation datasets (Fig. 7*E*).

### Using CRISPR screening data to explore potential HYP.SIG-related therapeutic targets

**Figure 8:**
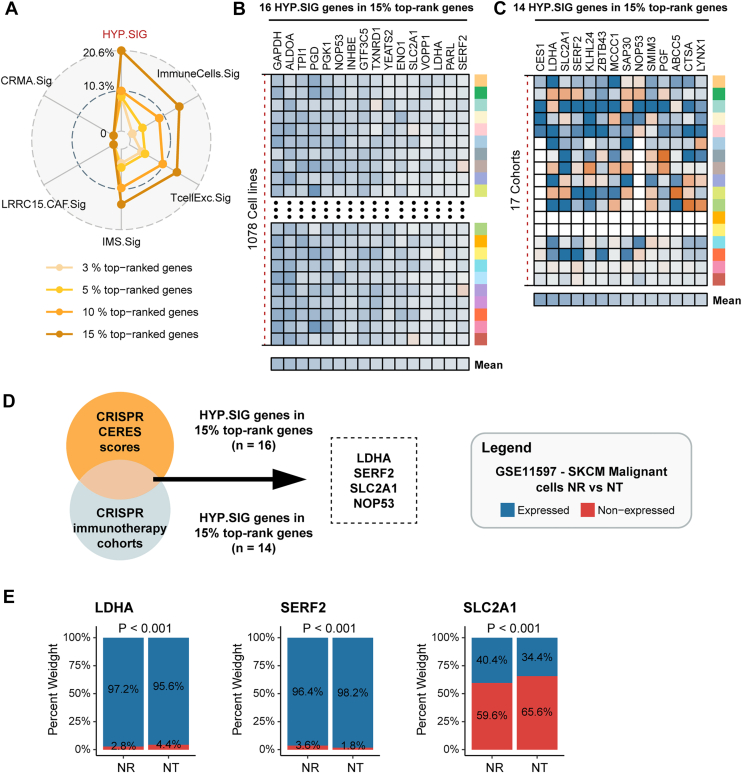
**Exploration of potential therapeutic targets from HYP.SIG**. *A*, *radar plot* shows the proportion of top-ranked genes overlapping with HYP.SIG and other signatures. *B* and *C*, heatmaps depict z scores of HYP.SIG genes in the 15% top-ranked genes in 1078 CRISPR cell lines and 17 CRISPR cohorts, respectively. *D*, the intersection of HYP.SIG genes in 15% top-rank genes across cell lines and immunotherapy cohorts was identified as potential therapeutic targets. *E*, *box plots* depict the difference of the number of the cells with expression of the specific gene and cells without expression of the gene between the patients with no response and patients with naïve treatment in malignant cells of GSE115978. The statistical difference was analyzed by the chi-square test. HYP.SIG, hypoxia-related transcriptomic signature.

We retrieved 1,078 CRISPR cell lines with CERES (CRISPR essentiality revealed by large-scale knockout screens) scores for 17,453 genes to estimate the dependency of the gene of interest in certain cancer cell line. The CERES score is a metric used to evaluate gene essentiality for cell survival. Genes with a low CERES score were interpreted as essential for the growth and survival of a given cancer cell line. Additionally, we collected 17 datasets derived from seven published CRISPR/Cas9 studies. These datasets evaluated the effect of 22,505 genes knockout on tumor immunity. We ranked each gene according to its mean z score. Genes with a low z-score were associated with resistance to the immunotherapy and may enhance anti-tumor immunity following knockout (Fig. S11, *A* and *B*). Subsequently, we determined the percentage of top-rank genes from HYP.SIG and previously established immune-resistance signatures in the CRISPR immunotherapy datasets. HYP.SIG had the dramatically higher percentage of top 3%, 5%, and 15% ranked genes than other signatures, except the top 10% ranked genes (HYP.SIG only revealed a slightly better percentage under this threshold) (Fig. 8*A*). In addition, there were 16 and 14 genes of HYP.SIG that were ranked in the top 15% in the CRISPR cell line data and the CRISPR immune response datasets, respectively (Fig. 8, *B* and *C*). Notably, four genes (*LDHA*, *SERF2*, *SLC2A1*, and *NOP53*) were present in both the two gene sets (Fig. 8*D*). We further evaluated the potential value of these genes in patient survival and the immune response across multiple cohorts (except *NOP53*, which lacks the expression profile). In the TCGA pan-cancer dataset, the expression of these three individual genes was significantly associated with overall survival (Fig. S12*A*). Additionally, analysis of cancer cell line data revealed that these genes were highly elevated in tumor cells under hypoxia, and only those with available expression profiles are presented (Fig. S12*B*). We then assessed the expression levels of target genes in the no response and naïve treatment groups in the GSE115978 scRNA-seq dataset. The percentage of cells expressing two of these genes was significantly higher in the NR group (Fig. 8*E*). Similarly, the expression levels of these genes in several immune cell types were higher under hypoxic conditions compared to normoxic conditions (Fig. S13). Based on these findings, we hypothesized that their effects on tumor growth and immune suppression might be induced by hypoxia (12, 32, 33). Overall, we identified four hub genes from HYP.SIG that affect cell growth and immune resistance employing CRISPR data. Knocking out these genes might impair tumor cell activity and enhance anti-tumor immunity. These critical genes could serve as promising therapeutic targets for immune checkpoint blockade therapy in tumor patients.

## Discussion

Prognostication of patient survival outcomes and prediction of immunotherapy response are critical for the development of personalized treatment strategies. Despite these breakthroughs, cancer immunotherapy still faces several challenges (8). With respect to efficacy, most patients are refractory to ICI therapy, making it difficult to predict patient responses. Mounting studies confirmed that the hypoxic microenvironment promotes cancer development in many aspects, especially therapy resistance (13, 34, 35). Cancer cells require lots of oxygen and nutrients to meet the energy demands of infinite proliferation and growth (36). However, the highly irregular distribution of neovascular network results in increased distances between the capillaries, and leaves gaps in oxygen delivery that foster tumor hypoxia (37). Meanwhile, reprogramming of glucose metabolism in tumor cells leads to an increase in oxygen consumption that further exacerbates tumor hypoxia (38). Hypoxia confers the advantages of proliferation, invasion, and metastasis on tumor cells, leading to more aggressive and treatment-resistant tumor phenotypes (39, 40). Moreover, hypoxia inhibits cytotoxic T cell function through modifications to both the tumor cells and tumor microenvironment, inducing immunosuppression and enabling tumor immune escape, thereby reducing the efficacy of ICI therapy and worsening patient prognosis (41). Although increasing lines of research have reported the underlying mechanisms between hypoxia and anti-tumor immunity and proposed new strategies for immunotherapy (10), the current status of hypoxia-targeted therapy is not encouraging (42). Moreover, most of the previously established pan-cancer predictive markers were developed based on immune system-related signatures in the RNA-seq analysis (43), whereas our findings overcome the limitation of lacking hypoxia-specific biomarkers. In this study, we proposed a cell-specific computational framework to identify more reliable predictive biomarkers, which would help to identify potential targets for clinical trials to maximize response rates and optimize patient selection.

We first conducted analyses in five single-cell RNA-seq cohorts assessing the degree of hypoxia in individual cell populations across multiple solid tumors based on a 15-gene expression signature. This gene set was shown to perform best in a study assessing the robustness of different hypoxia signatures. Interestingly, malignant cells exhibited the highest hypoxia status among all cell populations. Based on this finding, we established a four-step computational framework by integrating 38 public scRNA-seq datasets to generate a malignant cell-specific, HYP.SIG containing 68 genes. These genes were involved in a series of biological functions including angiogenesis, energy metabolism, cell proliferation, and DNA damage, which is in accordance with the previously reported hypoxia in the local microenvironment leads to increased reactive oxygen species production, oxidative DNA damage, and reprogramming of glucose metabolism and drives irregular angiogenesis (44, 45, 46, 47), thus promoting malignant behaviors such as proliferation, invasion and metastasis of cancer cells. Thereafter we calculated HYP.SIG scores and performed a large-scale comprehensive analysis. Primary tumor samples had higher HYP.SIG scores but greater variations in most cancer types, implying that tumor growth may be a process of adaptation to hypoxia. As mentioned in previous research, the HIF pathway could make cancer cells to evolve the capacity for oxygen-sensitive adaptive transcriptional responses and elicit metabolic reprogramming (48).

We evaluated the relationship of HYP.SIG to specific signaling pathways, immune function (including immune cell infiltration, immune checkpoint genes, and immune features), and patient survival (including PFS and OS). We found a significant positive correlation between HYP.SIG scores and multiple pro-tumorigenic signaling pathways in nearly all TCGA cohorts. Among these cellular pathways, mTOR and hypoxic signaling are interrelated in cancer cells (49). The mTOR gets environmental stimuli from hypoxia to bind to different upstream and downstream molecules, modulating cellular physiological and pathological processes. For instance, the PI3K-Akt-mTOR pathway, one of the most deregulated signaling pathways during tumorigenesis, controls cell growth, cell survival, and cell cycle progression (50). Moreover, mTOR signaling pathway is also a major driver of treatment resistance in a wide spectrum of malignant tumors (51). Regarding the immune cell response, tumors with high HYP.SIG demonstrated a decrease in cytotoxic immune cells such as CD8+ T cells, NK cells and M1 macrophages, whereas an increase in classical tumor-promoting immune cells like M2 macrophages. M1 macrophages are typically pro-inflammatory and have a high capacity to present antigens. They can inhibit cancer progression through mechanisms like the release of pro-inflammatory cytokines and the activation of immune responses. In contrast, M2 macrophages have anti-inflammatory properties and exhibit a poor ability to present antigens. They can suppress T-cell activity and lead to tumor immune escape (52). Consistent with these results, high HYP.SIG score was a major risk factor for both OS and PFS in a wide range of cancer types.

It is noteworthy that HYP.SIG positively correlated with TMB. It is well known that increased TMB is clinically associated with better ICI outcome (53, 54), contradicting our conclusion that tumors with high HYP.SIG presented with poor immune responses. The subgroup analysis indicated that tumors with low HYP.SIG displayed markedly better anti-tumor immunity regardless of TMB level, suggesting that hypoxia might contribute to immune resistance in high TMB tumors. Despite TMB is one of the most reliable predictive biomarkers for ICI, many patients with high TMB do not benefit from it. This further emphasizes the necessity of HYP.SIG as a predictive marker and provides a new perspective on the improvement of the current landscape of ICI therapy. HYP.SIG, as a hypoxia biomarker with significant clinical relevance, successfully predicted response to immunotherapy in nine ICI transcriptomic cohorts. Compared to previously published pan-cancer models for ICI therapy efficacy, which manifested excellent predictive power only in a few datasets, HYP.SIG exhibited more stable performance.

We identified genes with a strong correlation to overall patient survival at the pan-cancer level based on HYP.SIG and established a prognostic model. High risk scores demonstrated a strong positive correlation with advanced disease stage and impaired OS across multiple cancer types. To improve the model accuracy, we also developed a nomogram incorporating clinical disease stage. The results showed a high consistency between the survival prognostication based on the nomogram score and the actual survival probability over a 5-year period. We employed CRISPR data of cell lines and immune response cohorts to investigate potential drug targets from HYP.SIG. In total, we identified four hub genes that could be candidate therapeutic targets, including *LDHA*, *SERF2*, *SLC2A1*, and *NOP53*, which are critical for cell growth, survival, and immune resistance. As proof, LDHA-associated lactic acid production is a potent inhibitor of function and survival of T and NK cells, contributing to drug resistance (55, 56). *SLC2A1* affects tumor immune escape *via* inhibitory interactions with regulatory T cells (57). *SERF2* is of vital importance to cell growth, invasion, apoptosis, disease progression, and patient survival (58). Interestingly, *SERF2* exhibited a distinct prognostic pattern compared to other genes, showing a positive correlation with overall survival in the pan-cancer TCGA cohort. This opposite trend may reflect the biological complexity involving tumor-intrinsic features and the immune microenvironment (59). Specifically, the expression of *SERF2* in bulk RNA sequencing data may be influenced by immune cell infiltration or the presence of less aggressive tumor samples, which could alter its associations with clinical outcome. The study by Masanori Oshi *et al*. also demonstrated that tumor with an enhanced immune response is associated with more aggressive phenotype, but with better survival (60). Overall, our findings provide important clues to assess the characteristics and molecular mechanisms associated with cellular hypoxia across different cancer types and further guide the identification of novel biomarkers for predicting prognosis and immunotherapeutic response, which have great biomedical implications in future clinical applications.

## Experimental procedures

### Collection of mRNA-based hypoxia signatures

We comprehensively collected 10 hypoxia signatures among databases and published literature and selected Ye_HYPOXIA as a measure of cellular hypoxia (Tables S1 and S2). This gene signature was identified based on functional enrichment and *in vivo* co-expression analysis and was shown to perform better in reflecting hypoxia status.

### Pan-cancer single-cell RNA sequencing datasets

To generate a tumor-specific, HYP.SIG, 38 single-cell RNA-seq datasets were downloaded from the TISCH portal (http://tisch.comp-genomics.org/) (Table S3).

### Pan-cancer bulk sequencing transcriptomic datasets

Multi-omics data and clinically relevant information, including transcriptomic data, somatic mutation data, the stemness score (RNAss and DNAss), and homologous recombination deficiency-loss of heterozygosity, from TCGA and transcriptomic data from Genotype-Tissue Expression were derived from UCSC Xena website (https://xenabrowser.net/datapages/). MSI status were retrieved and downloaded from TCGA using R package ‘cBioPortalData’. Furthermore, diffuse large B cell lymphoma, acute myeloid leukemia, and thymoma were not considered in immune-related analyses because they primarily consist of immune cells.

Cancer types included for further analysis were as follows: adrenocortical carcinoma, BLCA, breast invasive carcinoma, CESC, cholangiocarcinoma, colon adenocarcinoma, lymphoid neoplasm diffuse large B-cell lymphoma, esophageal carcinoma, GBM, HNSC, kidney chromophobe, KIRC, kidney renal papillary cell carcinoma, acute myeloid leukemia, brain LGG, LIHC, LUAD, LUSC, mesothelioma, ovarian serous cystadenocarcinoma, PAAD, pheochromocytoma and paraganglioma, prostate adenocarcinoma, rectum adenocarcinoma, sarcoma, skin cutaneous melanoma (SKCM), stomach adenocarcinoma, testicular germ cell tumors, thyroid cancer, thymoma, UCEC, uterine carcinosarcoma, cveal melanoma.

### Collection of pan-cancer RNA-seq cohorts with ICI

To assess and validate the superior performance of HYP.SIG in predicting immunotherapy response for patients with various cancer types, we systemically collected 9 ICI RNA-Seq cohorts from the Gene Expression Omnibus and correspondingly published articles. The cohorts encompassed transcriptomics data as well as clinical information for five skin cutaneous melanoma cohorts (Hugo_SKCM_pre_aPD1, Liu_SKCM_pre_aPD1, Gide_SKCM_pre_aPD1, Riaz_SKCM_pre_aPD1, Van_SKCM_pre_aPD1), two urothelial carcinoma cohorts (Mariathasan_UC_pre_aPDL1, Snyder_UC_pre_aPDL1), one gastric cancer cohort (Kim_GC_pre_aPD1) and one renal cell carcinoma cohort (Bruan_RCC_pre_aPD1). Details of these ICI cohorts were shown in Table S4.

The five ICI cohorts with more patients and one ICI cohort with fewer patients were combined into one large dataset (n = 749), including Braun_RCC (n = 181), Mariathasan_UC (n = 348), Liu_SKCM (n = 121), Gide_SKCM (n = 73), Hugo_SKCM (n = 26). The ‘Combat’ algorithm in R package ‘sva’ was employed to remove batch effects. Then we used the ‘createDataPartition’ function in R package ‘Caret’ to randomly split this dataset into training (80%, n = 600) and validation sets (20%, n = 149). The other 4 ICI cohorts were combined into a external testing set (n = 155), including Riaz_SKCM (n = 49), Van_SKCM (n = 36), Kim_GC (n = 45), Snyder_UC (n = 25).

### Tumor cell line data

CERES dependency scores for 17,453 genes across 1078 cell lines, derived from genome-wide CRISPR/Cas9 screening studies, were downloaded from the DepMap portal (https://depmap.org/portal/). The score can be used to assess the importance of a gene to a particular cancer cell line, with a lower score indicating that the gene is more likely to influence the survival of a particular cell.

### Immune CRISPR screening datasets

To identify hub genes within HYP.SIG that could serve as potential therapeutic targets, we collected seven CRISPR-Cas9 screening datasets from published literature. Zhen *et al*. previously tidied up these studies and further categorized them into 17 datasets (Table S5). The study gauged the effect of 22,505 genes on tumor immunity, with a lower score indicating an improved immune response following gene knockout.

### The computational framework for identifying HYP.SIG

We utilized a four-step computational approach to identify a tumor-specific, hypoxia-related transcriptomic signature at the single-cell level. In the first step, we conducted GSVA to calculate hypoxia scores for individual patients based on Ye_HYPOXIA collected from the literature. Second, genes positively correlated with hypoxia in each individual dataset were regarded as ‘Gx’ (Spearman cor > 0, *p* < 0.05) by calculating Spearman correlations between gene expression levels and GSVA scores. Third, we identified genes that were differentially upregulated in malignant cells across datasets utilizing the ‘FindMarkers’ function in R package ‘Seurat’. Genes that were expressed in at least 10% of the cells of one or more cell types, with a log fold change greater than 0.25 and a *p*-value less than 0.05, were regarded as ‘Gy’. Genes fulfilling the requirements of the second and third steps were selected to generate Gn (n = 1–38) for each dataset. These gene sets are available online on GitHub (https://github.com/zzzzzzz0208/HYP.SIG). Finally, the geometric mean of Spearman correlation coefficients for each gene from ‘G1’ to ‘G38’ was calculated, and genes with a value greater than 0.4 were filtered to HYP.SIG, which ultimately contained a total of 68 genes (Table S6).

### Calculation of the HYP.SIG score

We described the R package ‘HypScore’, a computational method for assessing cellular hypoxia based on HYP.SIG in both bulk and single-cell gene expression profiles. If the input expression profile is derived from RNA-seq experiments, the user should select the ‘count’ class; if it consists of continuous counts, such as RNA-seq log-RPKMs or log-TPMs, the user should select the ‘integer’ class. Next, the R package ‘HypScore’ calculates a hypoxia score for each patient based on HYP.SIG using the GSVA algorithm. Meanwhile, we provided users with other nine known hypoxia gene sets as options. Finally, HypScore outputs a list that includes the user-selected gene set, hypoxia scores, and high- and low-hypoxia subgroups. The HypScore package is released under GitHub (https://github.com/zzzzzzz0208/HYP.SIG).

### Functional pathway enrichment analysis of HYP.SIG

To foster an in-depth comprehension of biological functions of genes in HYP.SIG, we performed enrichment analysis and visualized the results with adjusted *p*-value less than 0.05 using the R package ‘clusterProfiler’ and ‘ReactomePA’.

### Genomic variation analysis

The SCNA score for TCGA cancer types with at least samples was developed using GISTIC 2.0 to assess chromosome-, arm-, and focal-level alterations. For each tumor, alterations occurring in more than 70% of one chromosomal arm were defined as broad events. In the broad events, cases in which both chromosome arms exhibited the same copy number change were classified as chromosomal SCNA events, while all other cases were classified as arm SCNA events. Each event was defined as the following score based on its log2 copy number ratio from GISTIC2.0: 2 if the log2 ratio ≥ 1, one if the log2 ratio < 1 and ≥ 0.25, 0 if the log2 ratio < 0.25 and ≥−0.25, −1 if the log2 ratio < −0.25 and ≥−1, and −2 if the log2 ratio < −1. The absolute values of all focal-level scores in the tumor were summed into a focal score of a tumor. The arm- and chromosome-level score were calculated following the same process. Then we conducted rank-based normalization for SCNA scores at the three different levels separately within the same cancer type. We calculated the overall SCNA score of each tumor as the total of normalized focal-, arm-, and chromosome-level SCNA scores. Furthermore, events with the log2 ratio greater than 0.25 and less than −0.25 were defined as arm-level gains and less, respectively.

The total numbers of mutation events across TCGA cancer types were obtained using the R package ‘TCGAbiolinks’. The mutation data of LUAD were further visualized in the R package ‘maftools’.

### Immune infiltration analysis

The ESTIMATE algorithm was used to assess the levels of stromal cells, immune cells, and tumor purity. The CIBERSORT method was applied to estimate immune infiltration across TCGA samples based on transcriptomes deconvolution. The abundance of tissue-infiltrating immune and stromal cells was estimated by MCP counter based on gene expression.

### Construction of the HYP.SIG-related model for predicting response to ICI therapy

To predict the response to immunotherapy in cancer patients, we constructed a machine learning model in the training set by implementing the R package ‘Caret’. This model was based on six algorithms, including random forest (RF), Naïve Bayes (NB), AdaBoost Classification Trees (AdaBoost), k-nearest neighbors (KKNN), support vector machine (SVM), and boosted logistic regressions (LogiBoost). All these six machine learning algorithms were subjected to five times repeated 10-fold cross-validation to select the optimal parameter for the model. We subsequently tested and compared their performance in the validation set. The model with the highest AUC was chosen as the final predictive model for ICI response.

### Comparison of the performance of HYP.SIG with other pan-cancer predictive gene signatures

To assess the robustness of our model, we compared HYP.SIG with six other pan-cancer ICI response signatures in the combined and independent testing sets, including INFG, T cell-inflamed, PD-L1, LRRC15-CAF, NLRP3 inflammasome, and cytotoxic (Table S7). Codes and algorithms for the above six signatures were derived from previous research.

### Development of the HYP.SIG-related model for predicting the OS in patients

First, a total of 9637 samples in the TCGA pan-cancer cohort were included in our analysis after deleting samples without survival information. These samples were randomly divided into training (80%, n = 7710) and validation cohorts (20%, n = 1927) using the ‘createDataPartition’ function in R package ‘Caret’. In the TCGA pan-cancer cohort, we conducted least absolute shrinkage and selection operator, randomized survival prediction, and CoxBoost algorithms to identify 61, 30, and 60 significant features associated with patient survival among 68 HYP.SIG genes, respectively. The R package ‘glmnet’, R package ‘randomForestSRC’, and R package ‘CoxBoost’ were used to measure the importance of each gene for patient survival. We then constructed a multivariate Cox regression model with a stepwise method for predicting overall survival based on 29 common genes of the three algorithms in the TCGA pan-cancer training set. We calculated the HYP.SIG-related risk score per patient as follows: HYP.SIG-related risk score = Σ (regression coefficient) × (expression of each gene). Patients were split into high- and low-risk groups according to the median risk score, and significant differences in OS, DFI, disease-specific survival and PFI were determined *via* univariate Cox regression analysis and log-rank test. The R package ‘timeROC’ was implemented to calculate AUC values, which can be used to evaluate the sensitivity and specificity of the model. To intuitively manifest and visualize the performance of the relevant prognostic model, we also used R package ‘Survminer’ to draw a forest plot, a nomogram, and a time-dependent ROC curve.

### Statistical analysis

All statistical analyses in this study were conducted using R software (version 4.2.3) (https://www.r-project.org/). The Shapiro-Wilk test is used to tested for a normal distribution. The interquartile range method is used to test for statistical outliers. For datasets with small sample sizes or uncertain distributions, non-parametric tests were applied. The paired *t* test, Wilcoxon rank-sum test and Kruskal-Wallis test were employed to investigate differences between two subgroups or among more than two subgroups for continuous variables. The Chi-Square test was utilized to assess differences in categorical data. The Benjamini-Hochberg correction method was applied to obtain adjusted *p* values.

## Data availability

All data used in this study are publicly available and summarized in Table S10. The analytical pipeline, R scripts, and any relevant code are available online on GitHub (https://github.com/zzzzzzz0208/HYP.SIG).

## Supporting information

This article contains supporting information.

## Conflict of interest

The authors declare that they have no conflicts of interest with the contents of this article.
