# Supplementary material for: A cell-specific computational framework reveals a pan-cancer hypoxia signature predicting overall survival and ICI response
Source: J Biol Chem. 2025 Dec 17;302(2):111068. doi: 10.1016/j.jbc.2025.111068 (PMC12816908; doi:10.1016/j.jbc.2025.111068)
Supplement: Supplementary Figures [file mmc1.docx]

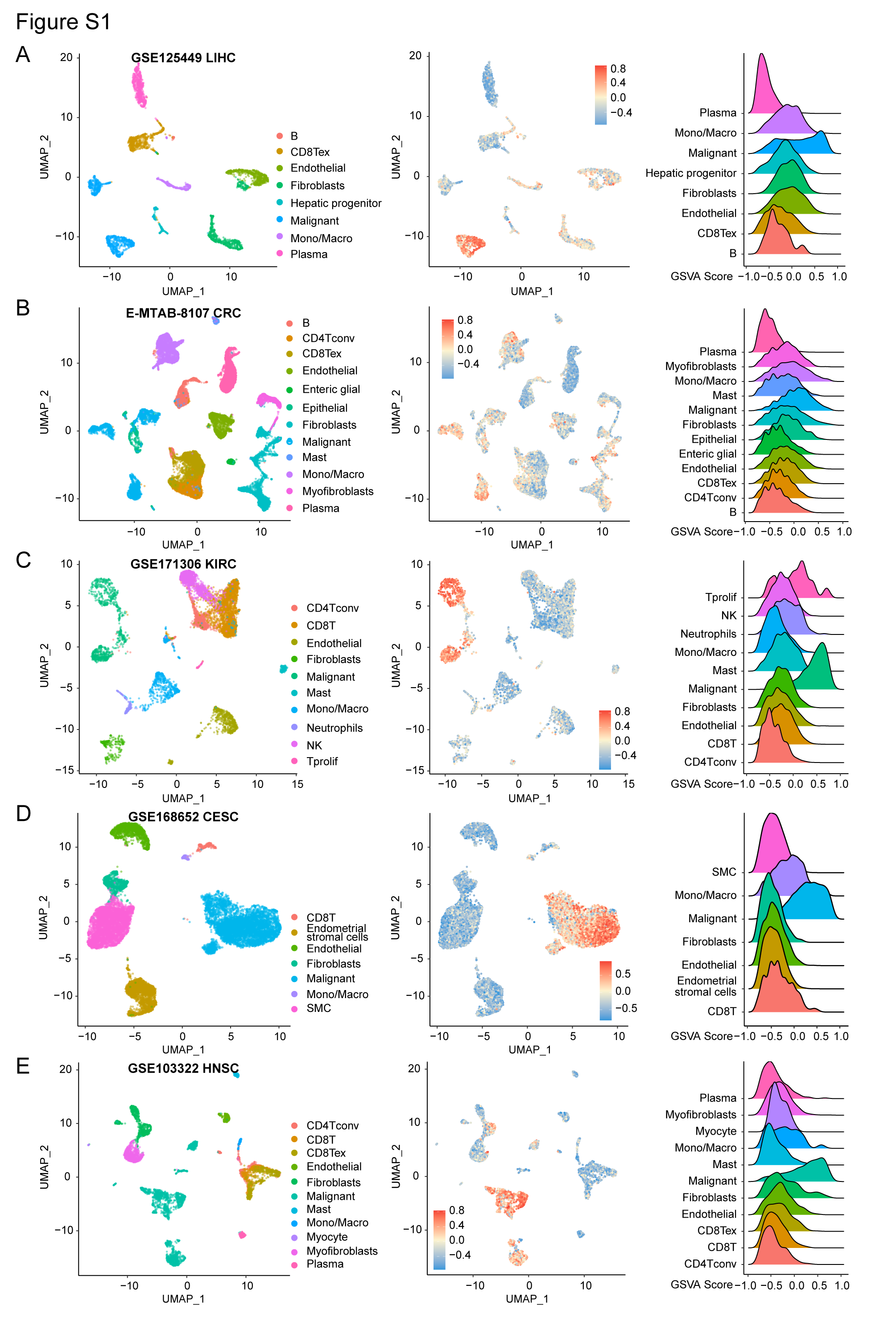


**Figure S1. Evaluation of the hypoxia status of different cell types in tumor microenvironment.**

Unified manifold approximation and projection (UMAP) plots depict the main cell types of five scRNA-seq datasets from various tumor entities, colored by cell type (**A-E**, left). Feature plots show the enrichment of hypoxia-related gene sets in individual cell clusters. Dark-reds indicates high hypoxia while dark-blue indicates low hypoxia (**A-E**, middle). Ridge plots visually show the distribution of the hypoxia score per cell (**A-E**, right).


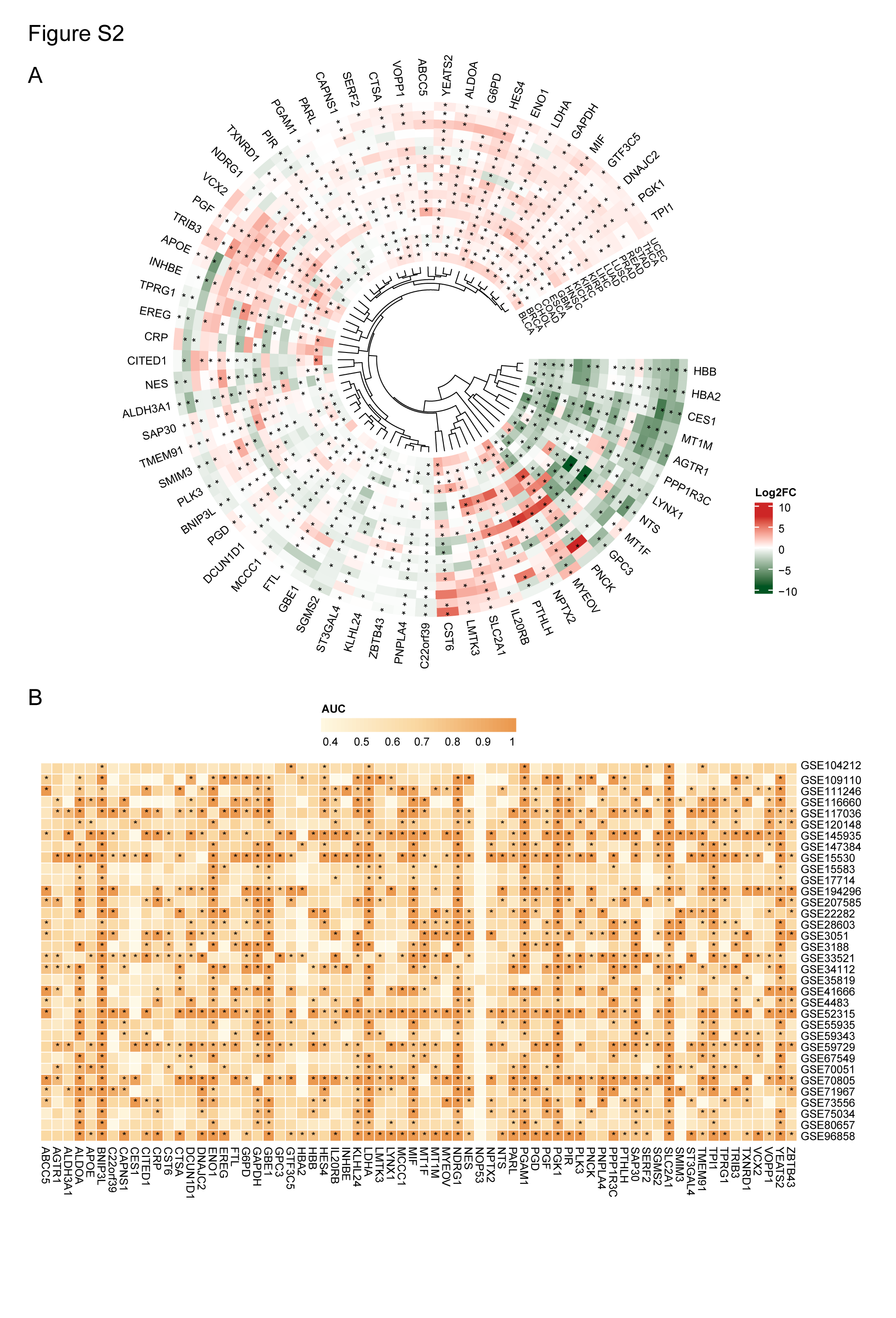


**Figure S2. Characterization of the 68-gene set.**

(**A**) Circular heatmap shows the differential expression of hypoxia signatures between tumor and normal samples across 18 cancer types. The statistical difference was analyzed by a paired t test, with * indicating a significant dysregulation (p < 0.05). (**B**) Hypoxia status was evaluated by expression levels of 68 genes. * indicates AUC > 0.7.


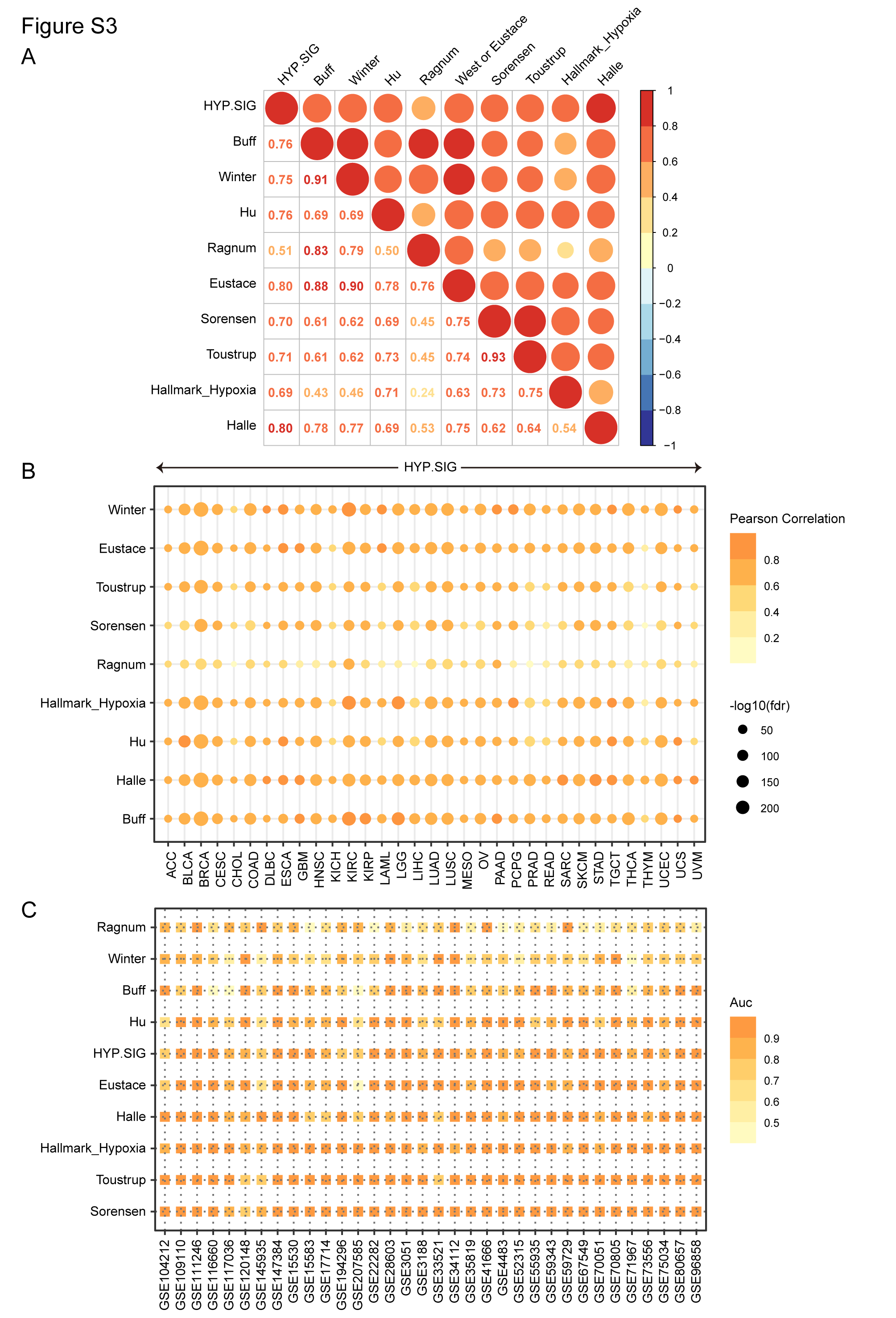


**Figure S3. Correlation of HYP.SIG and other hypoxia signatures.**

(**A** and **B**) Spearman correlations between HYP.SIG scores based on the 68-gene set and other gene sets from literature and MSigDB among TCGA tumor samples. (**C**) Hypoxia status was evaluated by hypoxia scores based on different gene sets. Color intensity indicates AUC values.


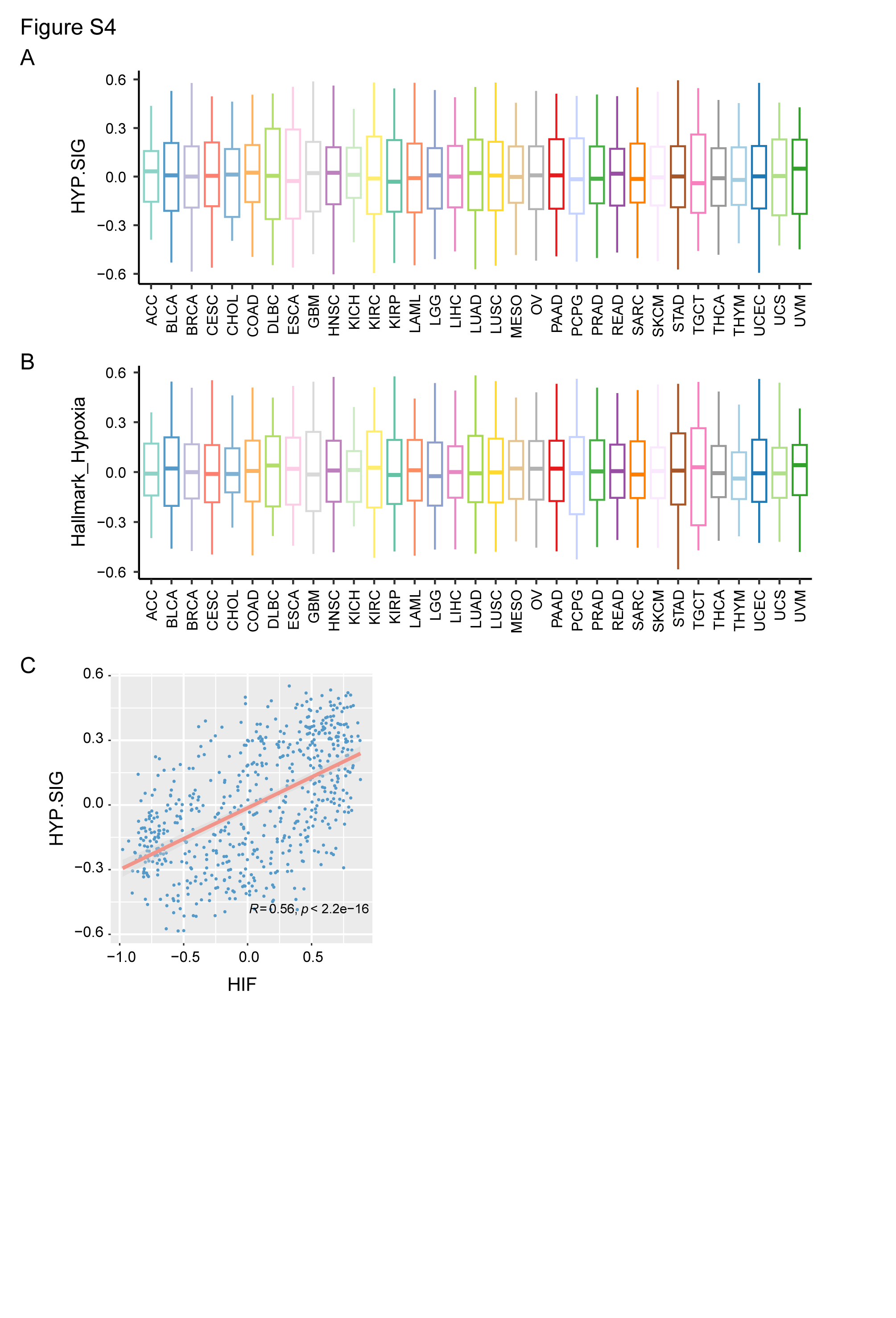


**Figure S4. Validation of HYP.SIG in KIRC.**

(**A** and **B**) Box plots show the hypoxia scores based on HYP.SIG (**A**) and Hallmark_Hypoxia (**B**) across TCGA cancer types. (**C**) Scatter plot shows the Spearman correlation of HYP.SIG and HIF targets in KIRC. GSVA scores were calculated to estimate the HYP.SIG and HIF scores per sample.


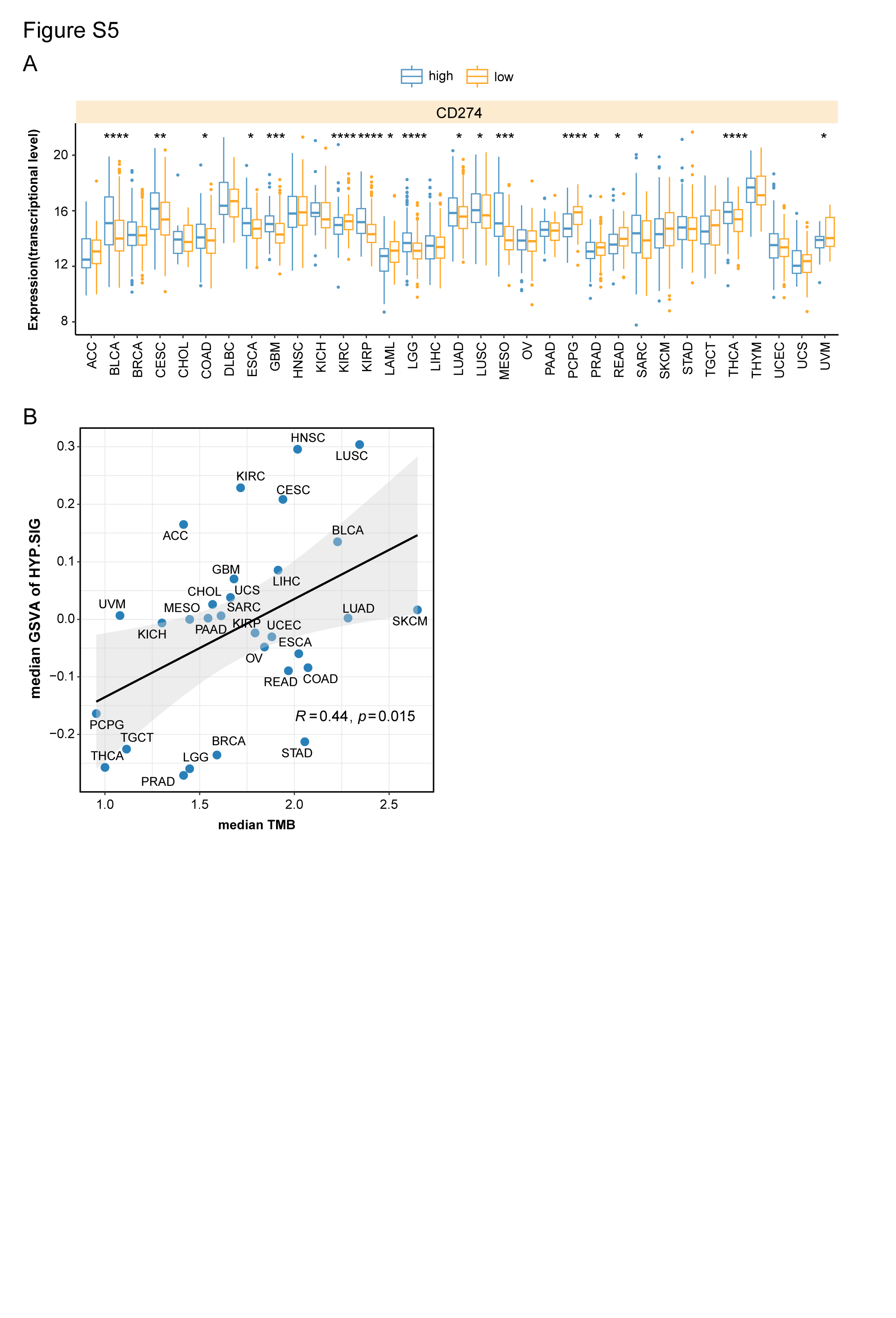


**Figure S5. Associations of hypoxia levels with tumor immunity.**

(**A**) Box plot shows the differences in CD274 expression between the high-HYP.SIG and low-HYP.SIG groups across cancer types. The statistical difference was analyzed by the Wilcoxon test, where * represents p < 0.05, ** represents p < 0.01, *** represents p < 0.001, **** represents p < 0.0001. (**B**) Scatter plot shows the Spearman correlation of median HYP.SIG and median TMB of individual cancer type in TCGA. GSVA scores were calculated to estimate the HYP.SIG score per sample.


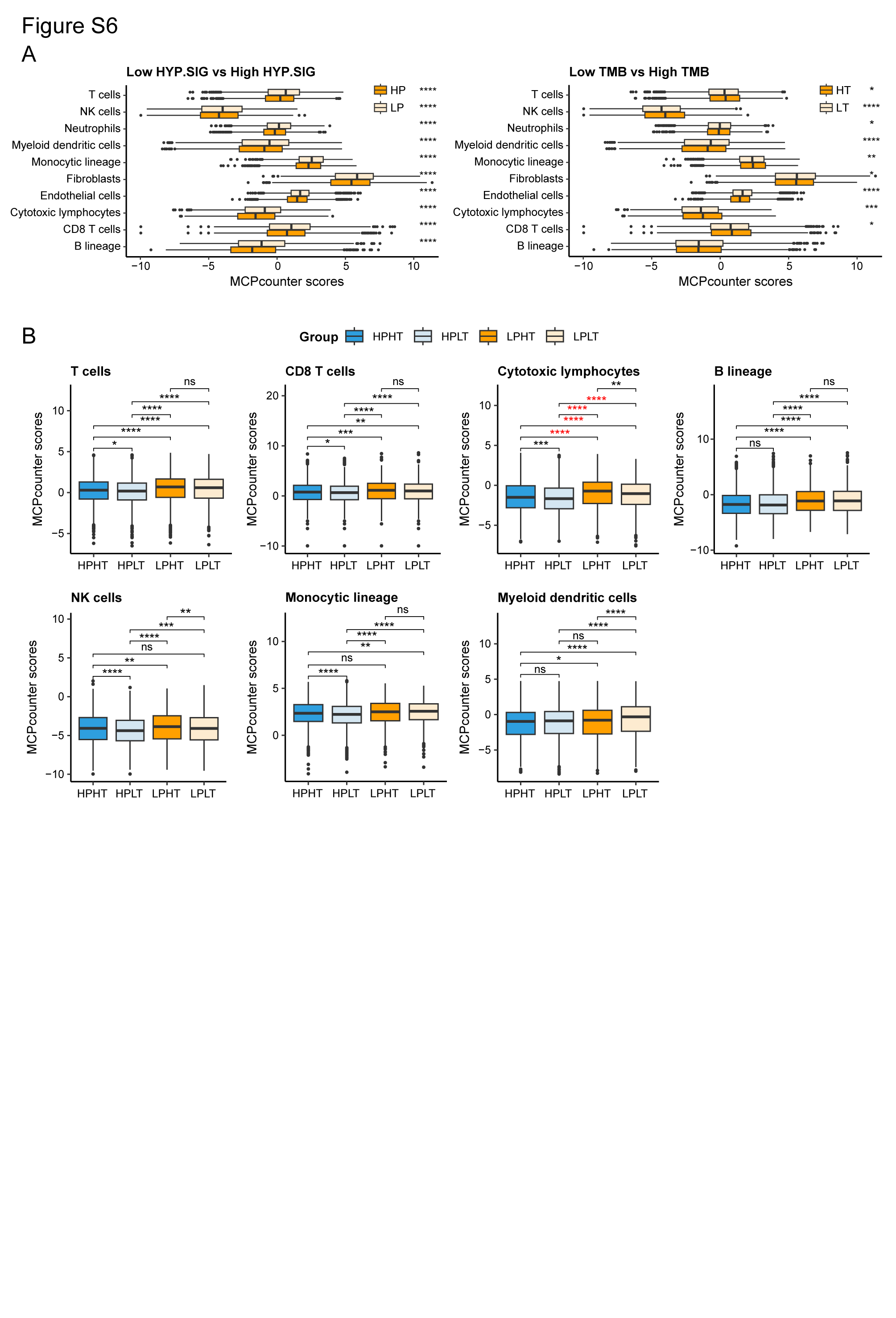


**Figure S6. Correlation of HYP.SIG and other hypoxia signatures.**

(**A**) Box plot shows the differences of immune cells calculated by MCP counter between high-HYP.SIG and low-HYP.SIG groups (left) and high-TMB and low-TMB groups (right). The statistical difference was analyzed by the Wilcoxon test, where * represents p < 0.05, ** represents p < 0.01, *** represents p < 0.001, **** represents p < 0.0001. (**B**) Box plot shows the difference of immune cell infiltration between four subgroups, including HPHT, HPLT, LPHT, and LPLT. The statistical difference was analyzed by the Wilcoxon test, where * represents p < 0.05, ** represents p < 0.01, *** represents p < 0.001, **** represents p < 0.0001.


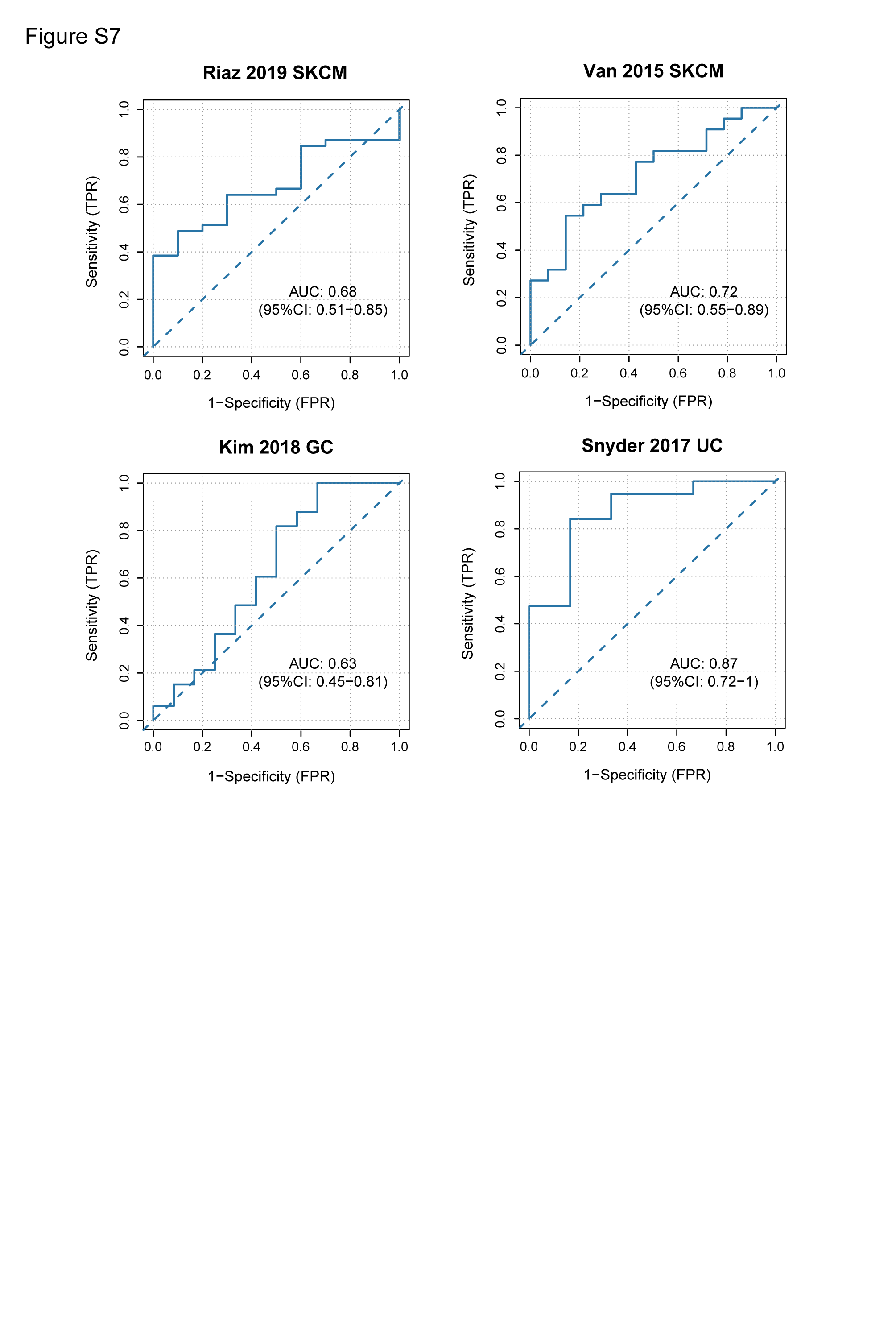


**Figure S7. The performance of the HYP.SIG-based predictive model in four individual testing cohorts.**

**
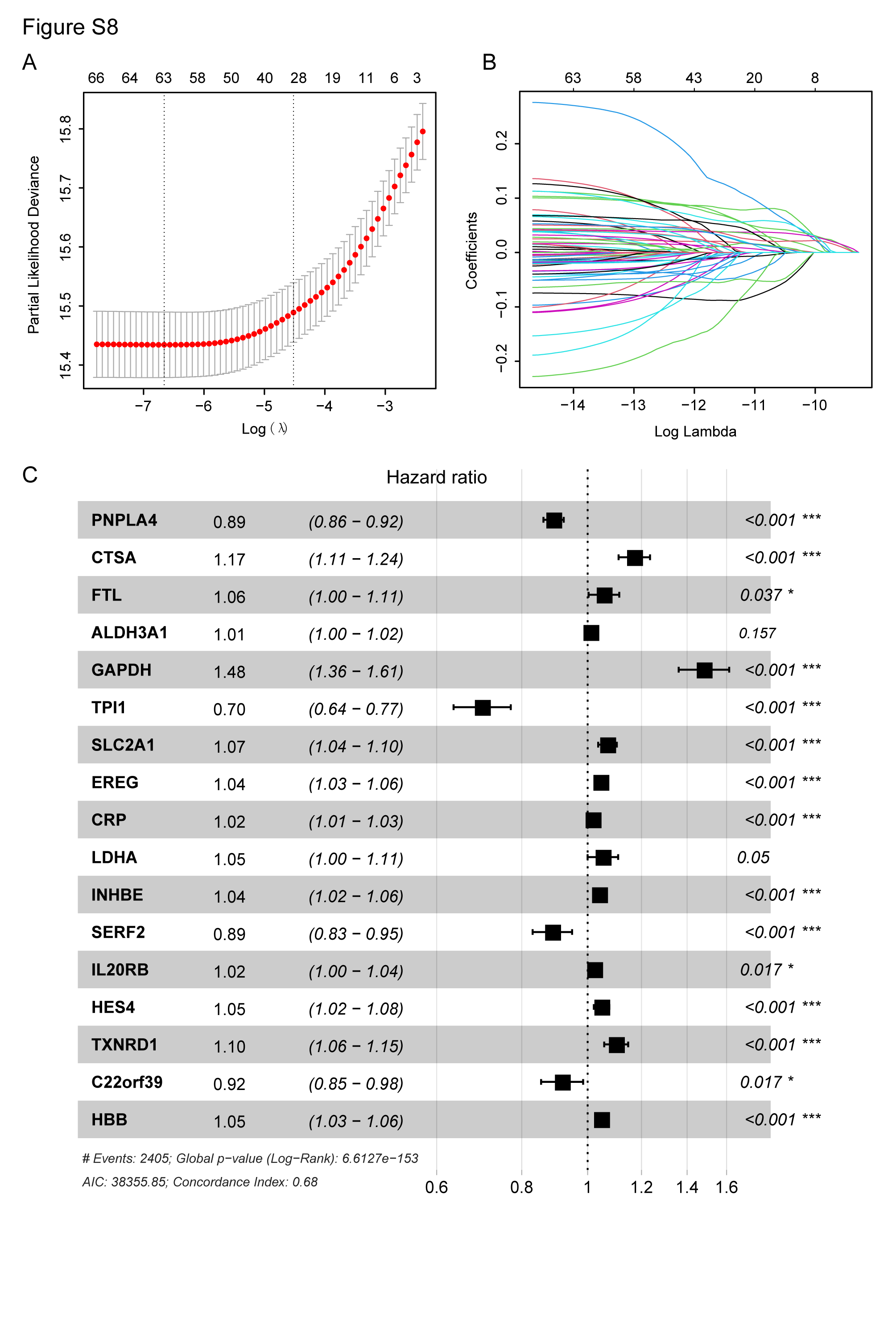
**

**Figure S8. Construction of the HYP.SIG-related prognostic model.**

(**A** and **B**) LASSO coefficient profiles of the 63 selected genes in HYP.SIG. 10-fold cross-validation to select tuning parameters for the LASSO model. (**C**) Forest plot of a multivariate Cox regression model in the overall survival of TCGA pan-cancer cohort.


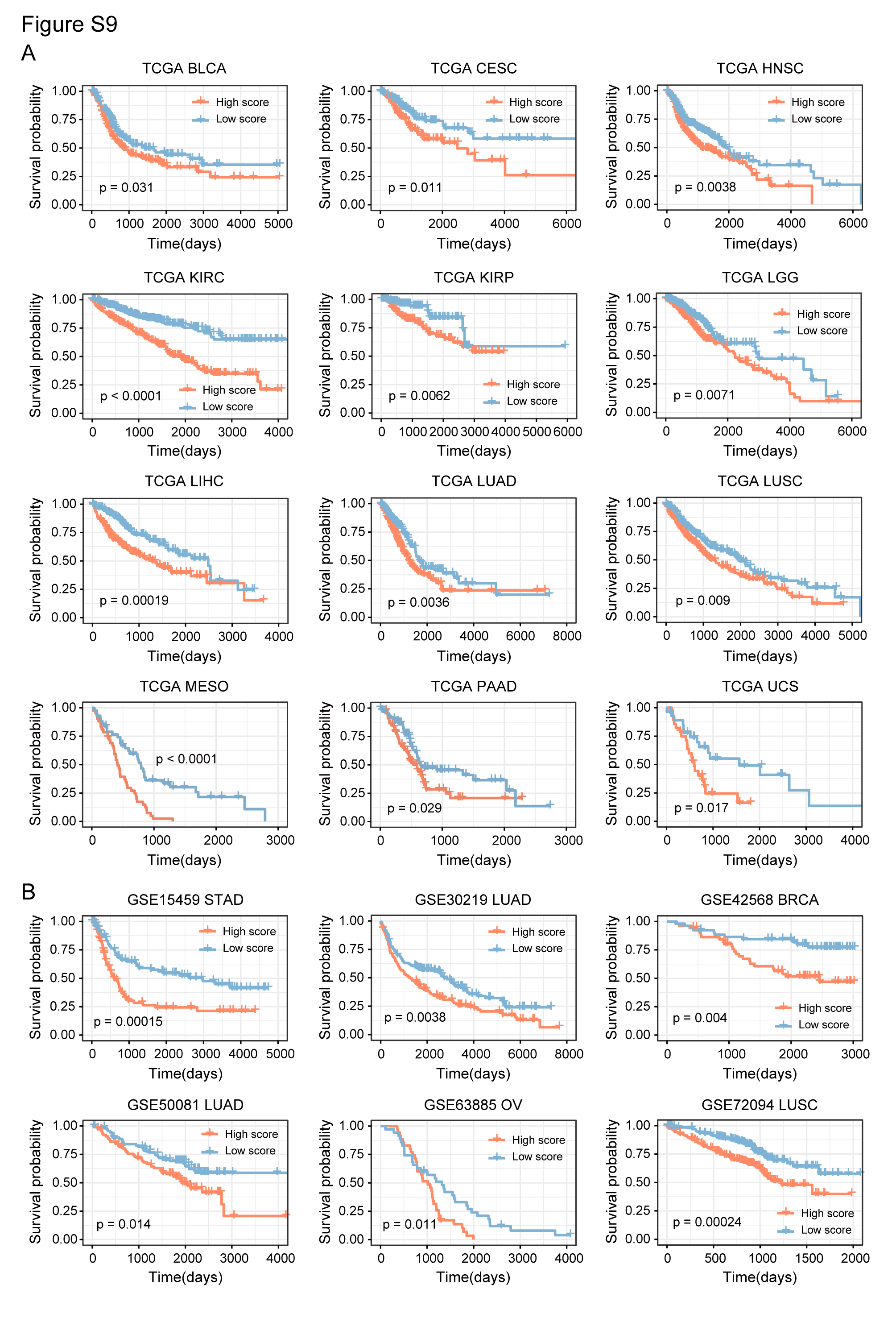


**Figure S9. The survival analysis in validation cohorts.**

(**A** and **B**) Kaplan-Meier curves compare the overall survival of high- and low-risk patients in a variety of TCGA and external cohorts, respectively.


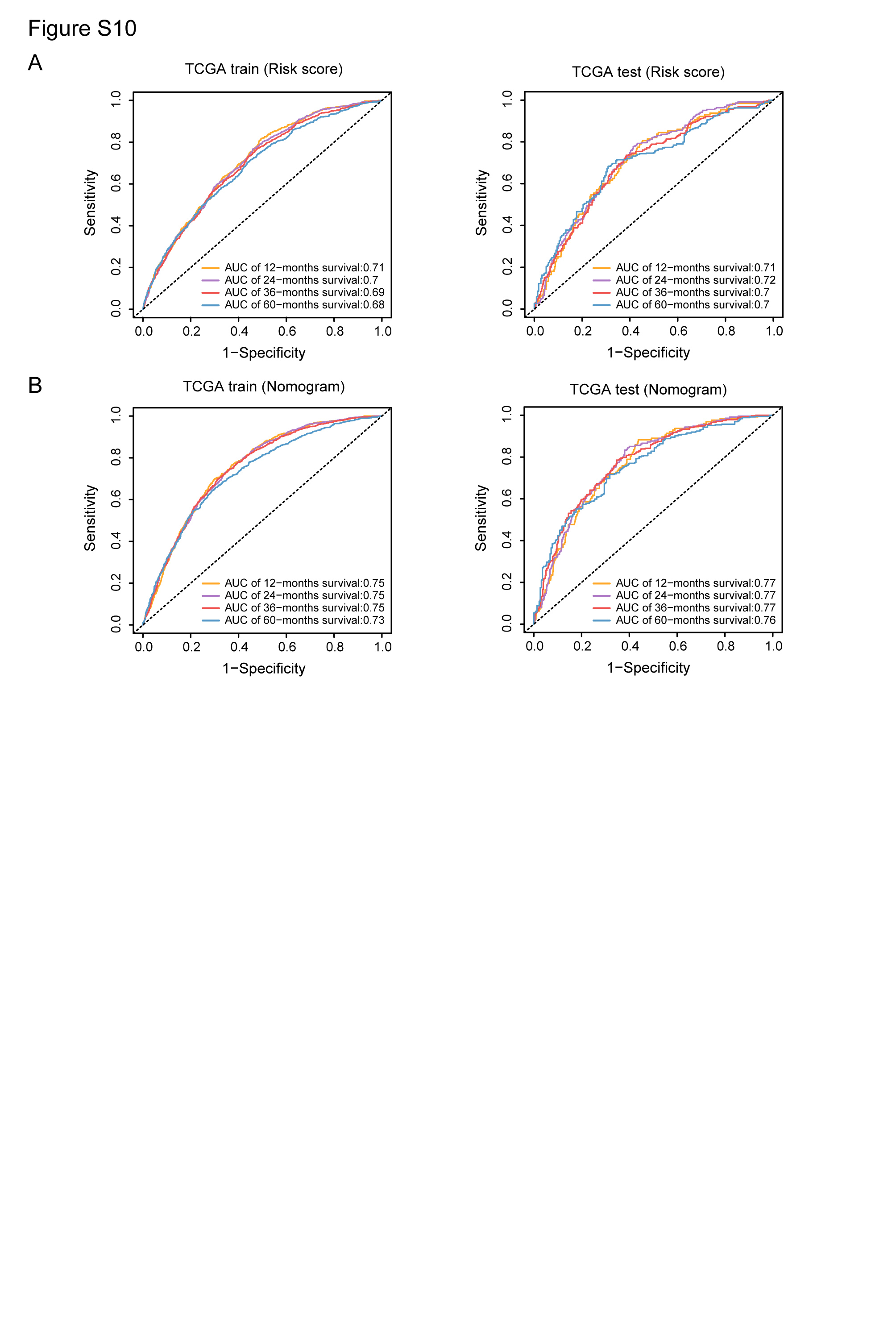


**Figure S10. The performance of a HYP.SIG-related prognostic model.**

(**A** and **B**) ROC curves depict the performance of HYP.SIG-related risk scores (**A**) and nomogram scores (**B**) in predicting overall survival of pan-cancer TCGA training and test cohorts.


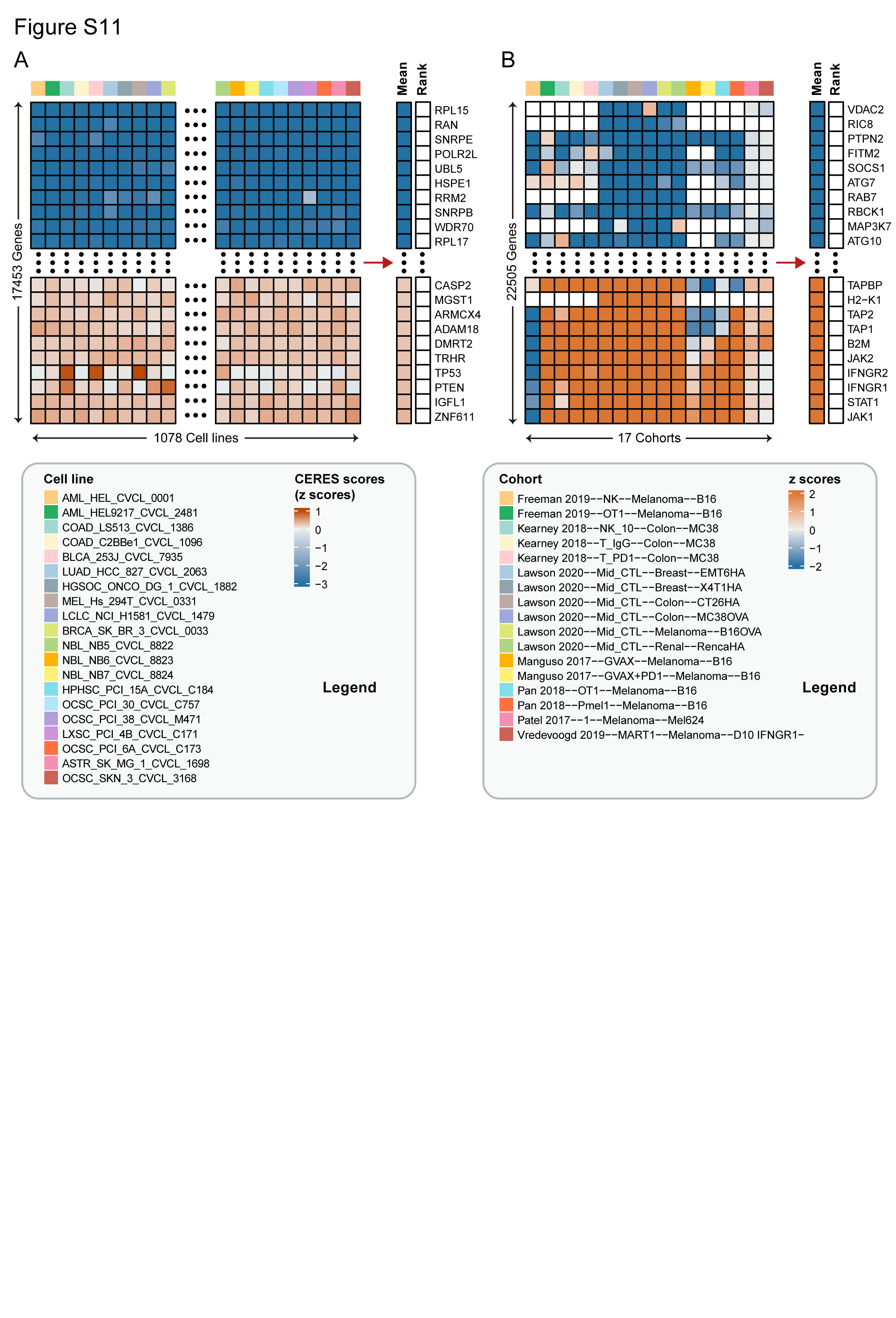


**Figure S11. Exploration of candidate therapeutic targets from HYP.SIG using CRISPR data.**

(**A**) Genes were ranked based on their average z scores, reflecting the effect of their knockout on cell growth across 1078 CRISPR cell lines. Top-ranking genes were more important for cell growth. (**B**) Genes were ranked based on their average z scores, reflecting the effect of their knockout on anti-tumor immunity across 17 CRISPR cohorts. Top‑ranking genes were more likely contribute to immune resistance. Blank squares indicate missing values.


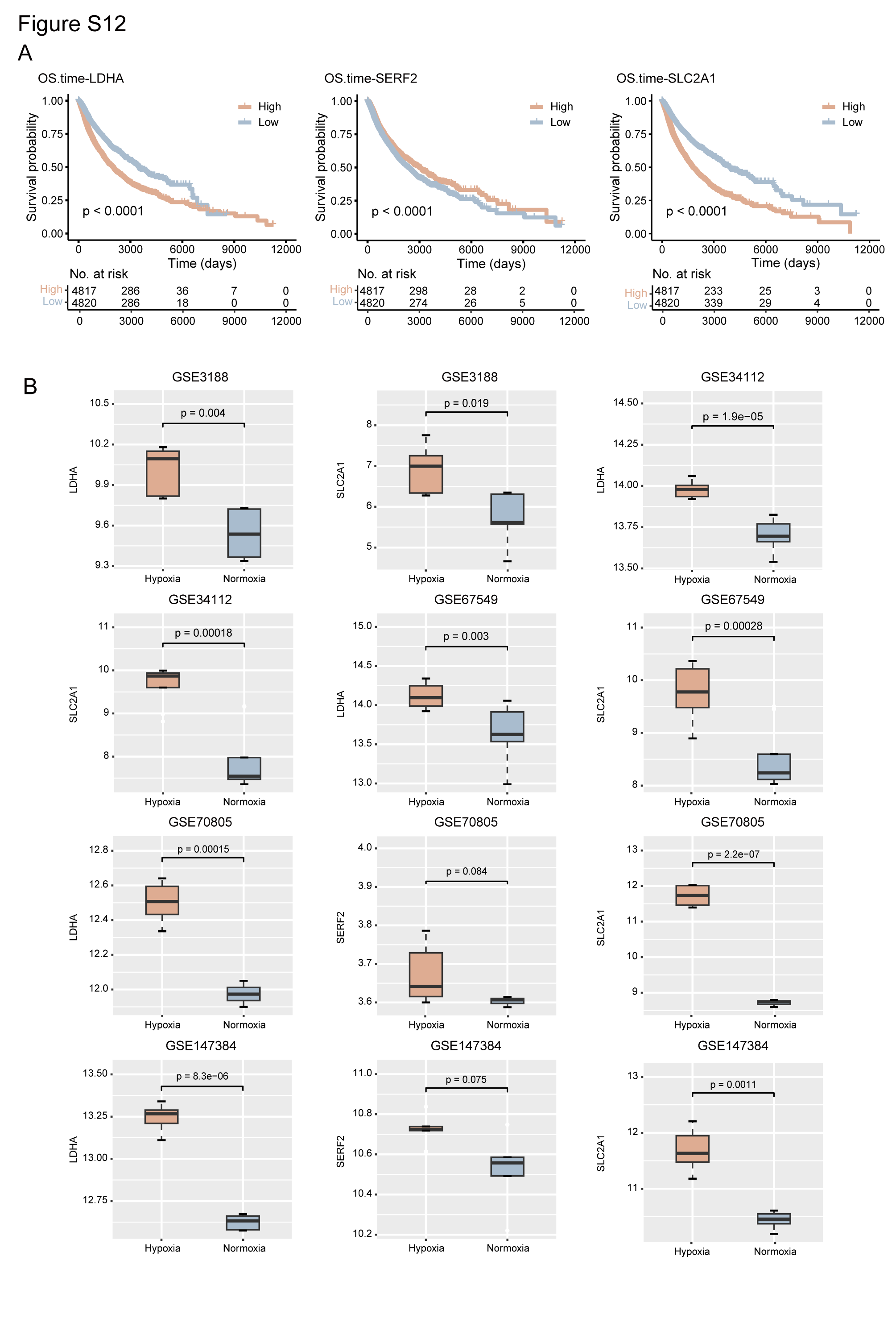


**Figure S12. Validation of the functional relevance of important targets identified from CRISPR cell line data.**
(**A**) Kaplan-Meier curves compare the overall survival of high- and low-expression groups in the TCGA pan-cancer cohort. (**B**) Box plot illustrates the expression levels (log2-transformed) of specific genes in hypoxia and normoxia samples across multiple cancer cell line datasets. Statistical difference was analyzed by a paired t test.


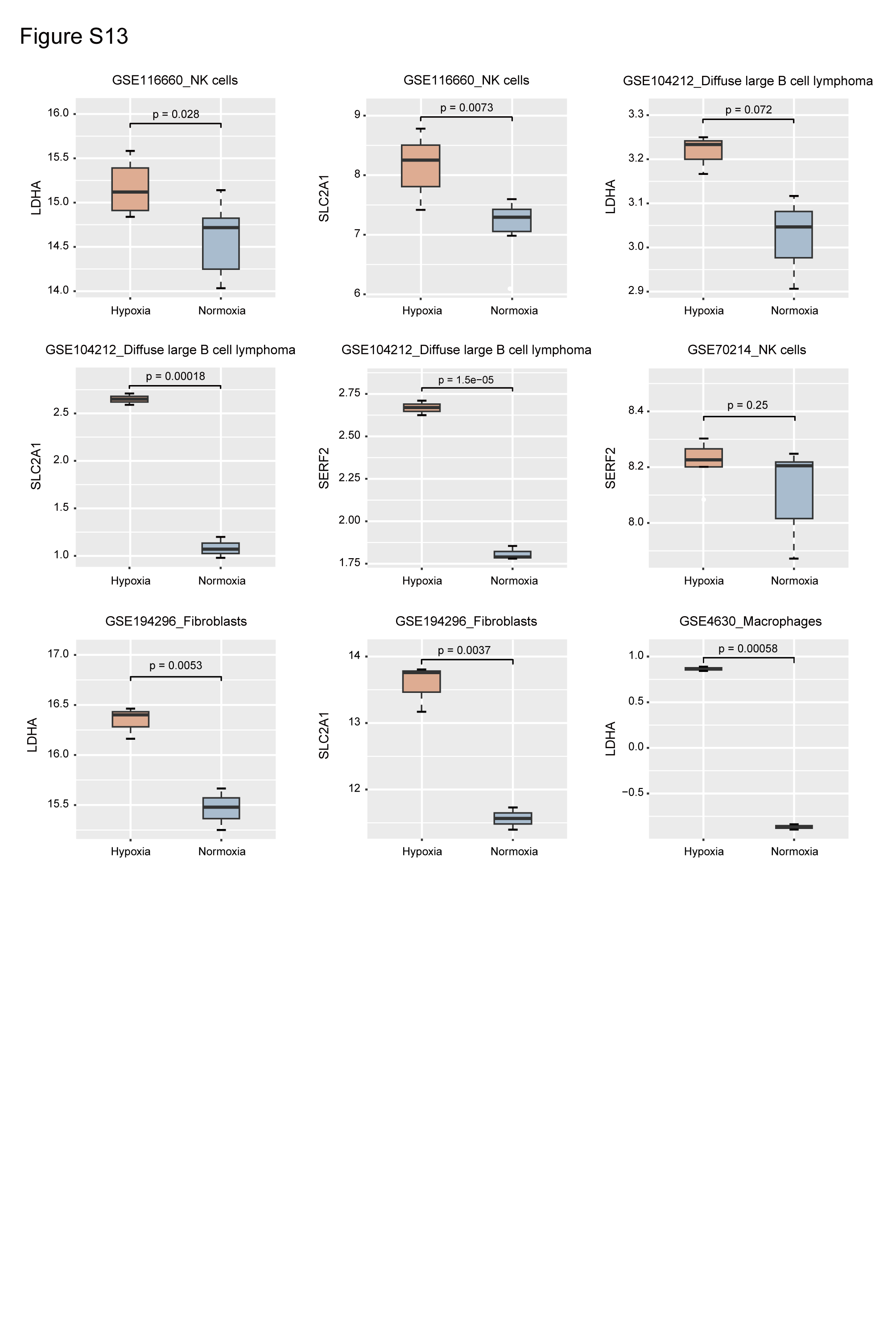


**Figure S13. Validation of the association between hypoxia and important targets identified from CRISPR immunotherapy datasets.**
