# Supplementary material for: A cell-specific computational framework reveals a pan-cancer hypoxia signature predicting overall survival and ICI response
Source: J Biol Chem. 2025 Dec 17;302(2):111068. doi: 10.1016/j.jbc.2025.111068 (PMC12816908; doi:10.1016/j.jbc.2025.111068)
Supplement: Supplementary Tables [file mmc2.docx]

**Supplement Tables**

**Table S1. Gene list of *Ye_HYPOXIA*.**

| VEGFA | TUBB6 | TPI1 | SLC2A1 | PGAM1 | P4HA1 | NDRG1 | MRPS17 |
| --- | --- | --- | --- | --- | --- | --- | --- |
| MRPS17 | LDHA | ENO1 | CDKN3 | ALDOA | ADM | ACOT7 |  |

**Table S2. List of 10 hypoxia-related gene signatures.**

| **Datasets ID** | **Number** | **Source** |
| --- | --- | --- |
| Buffa 2010 | 51 | PMID 20087356 |
| Halle 2012 | 31 | PMID 22890239 |
| Hu 2009 | 13 | PMID 19291283 |
| Ragnum 2014 | 32 | PMID 25461803 |
| Sorensen 2010 | 27 | PMID 20429727 |
| Toustrup 2012 | 15 | PMID 22385919 |
| Eustace 2013 | 26 | PMID 23820108 |
| Winter 2007 | 99 | PMID 17409455 |
| Ye 2019 (Ye_HYPOXIA) | 15 | PMID 31984309 |
| HALLMARK_HYPOXIA | 200 | MsigDB |

**Table S3. List of scRNA datasets applied to develop HYP.SIG**

| **Datasets** | **Cancer** | **Number**  **(patients)** | **Number**  **(cells)** | **Platform** | **PMID** |
| --- | --- | --- | --- | --- | --- |
| GSE132509 | ALL | 11 | 37936 | 10xGenomics | 32415257 |
| GSE116256 | AML | 21 | 38348 | Smart-seq2 | 30827681 |
| GSE123813 | BCC | 11 | 52884 | 10xGenomics | 31359002 |
| EMTAB8107 | BRCA | 14 | 33043 | 10xGenomics | 32561858 |
| GSE143423 | BRCA | 2 | 4375 | 10xGenomics | [bioRxiv](https://www.biorxiv.org/content/10.1101/2019.12.30.890517v1) |
| GSE148673 | BRCA | 6 | 10359 | 10xGenomics | 33462507 |
| SRP114960 | BRCA | 8 | 2472 | SNRS | 29681456 |
| GSE168652 | CESC | 1 | 22998 | 10xGenomics | 33996252 |
| GSE125449 | CHOL | 10 | 5761 | 10xGenomics | 31588021 |
| GSE138709 | CHOL | 5 | 33990 | 10xGenomics | 32505533 |
| EMTAB8107 | CRC | 7 | 23176 | 10xGenomics | 32561858 |
| GSE146771 | CRC | 10 | 10468 | Smart-seq2 | 32302573 |
| GSE166555 | CRC | 12 | 66050 | 10xGenomics | 34409732 |
| GSE138794 | GBM | 9 | 18458 | 10xGenomics | 31554641 |
| GSE139448 | GBM | 3 | 12152 | 10xGenomics | 32004492 |
| GSE141982 | GBM | 2 | 5263 | 10xGenomics | 32105316 |
| GSE103322 | HNSC | 18 | 5902 | Smart-seq2 | 29198524 |
| GSE159115 | KICH | 1 | 2850 | 10xGenomics | 34099557 |
| GSE159115 | KIRC | 8 | 27669 | 10xGenomics | 34099557 |
| GSE171306 | KIRC | 2 | 11427 | 10xGenomics | 34168986 |
| GSE125449 | LIHC | 9 | 3834 | 10xGenomics | 31588021 |
| EMTAB6149 | NSCLC | 5 | 40218 | 10xGenomics | 29988129 |
| GSE117570 | NSCLC | 4 | 11453 | 10xGenomics | 31033233 |
| GSE127465 | NSCLC | 7 | 31179 | Smart-seq2 | 30979687 |
| GSE143423 | NSCLC | 3 | 12193 | 10xGenomics | [bioRxiv](https://www.biorxiv.org/content/10.1101/2019.12.30.890517v1) |
| EMTAB8107 | OV | 5 | 24781 | 10xGenomics | 32561858 |
| GSE118828 | OV | 9 | 1901 | Smart-seq2 | 30383866 |
| CRA001160 | PAAD | 35 | 57443 | 10xGenomics | 31273297 |
| GSE111672 | PAAD | 3 | 6122 | 10xGenomics | 31932730 |
| GSE162708 | PAAD | 1 | 22133 | 10xGenomics | 34671197 |
| GSE137829 | PRAD | 6 | 8640 | 10xGenomics | 33328604 |
| GSE141445 | PRAD | 13 | 33441 | 10xGenomics | 33420488 |
| GSE143791 | PRAD | 16 | 36850 | 10xGenomics | 34719426 |
| GSE176031 | PRAD | 11 | 18807 | Drop-seq | 35013146 |
| GSE115978 | SKCM | 31 | 7186 | Smart-seq2 | 30388455 |
| GSE72056 | SKCM | 19 | 4645 | Smart-seq2 | 27124452 |
| GSE134520 | STAD | 13 | 41554 | 10xGenomics | 31067475 |
| GSE139829 | UVM | 11 | 103703 | 10xGenomics | 31980621 |
| **Total** | **38** | **362** | **893464** |  |  |

**Table S4. List of immunotherapy cohorts utilized in this study.**

| **Cohorts**  **(Training and Validation)** | **Cancer** | **Treatment** | **Number**  **(patients)** | **PMID** |
| --- | --- | --- | --- | --- |
| Braun  2020 RCC | Renal cell carcinoma | aPD-1 | 181 | 32472114 |
| Mariathasan  2018 UC | Urothelial carcinoma | aPD-1 | 348 | 29443960 |
| Hugo  2016 SKCM | Melanoma | aPD-1 | 26 | 26997480 |
| Liu  2019 SKCM | Melanoma | aPD-1 | 121 | 31792460 |
| Gide  2019 SKCM | Melanoma | aPD-1 / Combo | 73 | 30753825 |
| **Total**  **(Training and Validation)** |  |  | **749** |  |
| **Cohorts (Independent Testing)** | **Cancer** | **Treatment** | **Number**  **(patients)** | **PMID** |
| Riaz  2017 SKCM | Melanoma | aPD-1 | 49 | 29033130 |
| Van  2015 SKCM | Melanoma | aCTLA-4 | 36 | 26359337 |
| Kim  2018 GC | Gastric cancer | aPD-1 | 45 | 30013197 |
| Snyder  2017 UC | Urothelial carcinoma | aPD-1 | 25 | 28552987 |
| **Total**  **(Independent Testing)** |  |  | **155** |  |
| **Total**  **(overall cohorts)** |  |  | **904** |  |

**Table S5. List of 17 immunotherapy cohorts with CRISPR.**

| **CRISPR**  **datasets** | **Cell ID** | **Cancer** | **PMID** |
| --- | --- | --- | --- |
| Freeman 2019 | Freeman 2019--NK--Melanoma--B16 | Melanoma | 31509742 |
| Freeman 2019 | Freeman 2019--OT1--Melanoma--B16 | Melanoma | 31509742 |
| Kearney 2018 | Kearney 2018--NK_10--Colon--MC38 | Colon | 26776993 |
| Kearney 2018 | Kearney 2018--T_IgG--Colon--MC38 | Colon | 26776993 |
| Kearney 2018 | Kearney 2018--T_PD1--Colon--MC38 | Colon | 26776993 |
| Lawson 2020 | Lawson 2020--Mid_CTL--Breast--  EMT6HA | Breast | 32968282 |
| Lawson 2020 | Lawson 2020--Mid_CTL--Breast--  X4T1HA | Breast | 32968282 |
| Lawson 2020 | Lawson 2020--Mid_CTL--Colon--  CT26HA | Colon | 32968282 |
| Lawson 2020 | Lawson 2020--Mid_CTL--Colon--  MC38OVA | Colon | 32968282 |
| Lawson 2020 | Lawson 2020--Mid_CTL--Melanoma-  -B16OVA | Melanoma | 32968282 |
| Lawson 2020 | Lawson 2020--Mid_CTL--Renal--  RencaHA | Renal | 32968282 |
| Manguso 2017 | Manguso 2017--GVAX--Melanoma--  B16 | Melanoma | 28723893 |
| Manguso 2017 | Manguso 2017--GVAX+PD1--  Melanoma--B16 | Melanoma | 28723893 |
| Pan 2018 | Pan 2018--OT1--Melanoma--B16 | Melanoma | 29301958 |
| Pan 2018 | Pan 2018--Pmel1--Melanoma--B16 | Melanoma | 29301958 |
| Patel 2017 | Patel 2017--1--Melanoma--Mel624 | Melanoma | 28783722 |
| Vredevoogd 2019 | Vredevoogd 2019--MART1--  Melanoma--D10 IFNGR1- | Melanoma | 31303383 |

**Table S6. Gene list of *HYP.SIG*.**

| PGD | ALDOA | LDHA | APOE | HBA2 | ENO1 | CES1 | PARL |
| --- | --- | --- | --- | --- | --- | --- | --- |
| TPI1 | G6PD | PGAM1 | ALDH3A1 | CST6 | AGTR1 | NTS | TPRG1 |
| LMTK3 | PNCK | SERF2 | C22orf39 | LYNX1 | HBB | GPC3 | VCX2 |
| PGK1 | MCCC1 | GAPDH | PPP1R3C | NDRG1 | TRIB3 | MIF | NPTX2 |
| SLC2A1 | MT1F | SAP30 | ST3GAL4 | ABCC5 | HES4 | GBE1 | PTHLH |
| SMIM3 | FTL | MYEOV | GTF3C5 | IL20RB | PLK3 | INHBE | VOPP1 |
| CTSA | TMEM91 | PNPLA4 | DNAJC2 | MT1M | YEATS2 | TXNRD1 | SGMS2 |
| ZBTB43 | PGF | CAPNS1 | CRP | NES | EREG | CITED1 | NOP53 |
| KLHL24 | DCUN1D1 | BNIP3L | PIR |  |  |  |  |

**Table S7. List of predictive pan-cancer gene expression signatures for immunotherapy.**

| **Pan-cancer signatures** | **Algorithms** | **Description** |
| --- | --- | --- |
| IFNG.Sig | Average gene expression | An IFN-gamma-related profile that predicts PD-1blockade efficacy |
| T.cell.inflamed.Sig | Average gene expression | T-cell inflamed gene expression profiles |
| PD-L1.Sig | Expression of PD-L1 | Gene expression profiles of PD-L1 / PDCD1 |
| LRRC15.CAF.Sig | eigenWeightedMean  (R package *multiGSEA* *v1.1.99*) | Gene expression signature of LRRC15+ Cancer associated fibroblast |
| Cytotoxic.Sig | Geometric mean of gene expresssion | Genes associated with cytotoxic activities |
| NLRP3.Sig | ssGSEA | NLRP3 inflammasome-related genes derived from pan-cancer analysis |

**Table S8. List of independent datasets utilized to validate the performance and assess the robustness of *HYP.SIG*.**

| **Datasets** | **Description** | **Number**  **(patients)** |
| --- | --- | --- |
| GSE104212 | Human lymphoma cell lines HLY-1 and SUDHL2 | 12 |
| GSE109110 | Tumor-associated macrophages in pancreatic cancer | 12 |
| GSE111246 | MCF7 breast cancer cell lines | 15 |
| GSE116660 | NK cells of healthy donors | 12 |
| GSE117036 | A549 NSCLC cell lines | 12 |
| GSE120148 | iPSCs grown in hypoxia and normoxia cell culture conditions | 22 |
| GSE145935 | Hypoxia-induced astrocytes in vitro | 6 |
| GSE147384 | HeLa and SiHa cervical cancer cell lines | 20 |
| GSE15530 | MCF7 breast cancer cell lines | 8 |
| GSE15583 | Neuroblastoma cells | 18 |
| GSE17714 | Neuroblastoma cells | 22 |
| GSE188608 | 3D in vitro model JMML-patients cells | 10 |
| GSE194296 | Skin fibroblasts | 6 |
| GSE207585 | Myeloma cell lines | 8 |
| GSE22282 | Blood monocytes isolated from healthy volunteers | 6 |
| GSE28603 | Simpson-Golabi-Behmel syndrome adipocytes | 12 |
| GSE3051 | HeLa cervical cancer cell lines | 6 |
| GSE3188 | MCF7 breast cancer cell lines | 10 |
| GSE33521 | HeLa cervical cancer cell lines | 4 |
| GSE34112 | PC3 prostate cancer cells | 16 |
| GSE35819 | Human embryonic stem  cell lines | 18 |
| GSE41666 | HCT116 colon carcinoma and HepG2 hepatocellular carcinoma cells | 12 |
| GSE4483 | Astrocyte and Hela cells | 12 |
| GSE52315 | MM1S multiple myeloma cell lines | 6 |
| GSE52795 | Human oral cancer cell lines | 6 |
| GSE55935 | 22Rv1, LNCaP, PC-3 and DU145 human prostate cancer cell lines | 8 |
| GSE59343 | Human melanoma cell lines | 24 |
| GSE59729 | Huh-7 hepatocarcinoma cell lines | 24 |
| GSE67549 | Pancreatic cancer cell lines | 18 |
| GSE70051 | HNSCC and cervical cell lines | 32 |
| GSE70805 | MCF7 breast cancer cell lines | 9 |
| GSE71967 | Human umbilical vein endothelial cells | 30 |
| GSE73556 | Glioma initiating cells | 6 |
| GSE75034 | Hela, SW756, C-33, C-41, ME-180, and HT-3 cervical cancer cell lines | 12 |
| GSE77606 | MDA-MB-231, SK-BR-3 and MCF7 breast cancer, A549, PC9 and LCAI lung cancer, MESO1 mesothelioma, HT29, SW48 and Caco2 colon cancer cell lines | 18 |
| GSE79069 | HeLa cervical cancer cell lines | 12 |
| GSE80545 | Myeloma patient samples | 8 |
| GSE80657 | DU145, PC-3 and 22Rv1 prostate cancer cells | 36 |
| GSE96858 | U266 myeloma cell lines | 6 |
| **Total** |  | **534** |

**Table S9. List of 75 immune checkpoint genes.**

| HLA-DQB1 | MICA | HLA-A | HLA-DQB2 | MICB | HLA-B |
| --- | --- | --- | --- | --- | --- |
| HLA-DPA1 | HLA-DQA1 | HLA-DRB1 | HLA-DPB1 | HLA-DRA | HLA-DQA2 |
| HLA-C | HLA-DRB5 | ICAM1 | ITGB2 | SELP | BTN3A2 |
| C10orf54 | SLAMF7 | PDCD1LG2 | CD276 | BTN3A1 | CD274 |
| VTCN1 | CD80 | ICOSLG | CD28 | VEGFB | TGFB1 |
| CCL5 | IFNA1 | CD70 | IL4 | TNFSF4 | IFNA2 |
| IL2 | IL10 | CX3CL1 | TNFSF9 | TNF | IL12A |
| IFNG | CXCL9 | IL13 | IL1A | CD40LG | IL1B |
| CXCL10 | VEGFA | ARG1 | GZMA | PRF1 | ENTPD1 |
| HMGB1 | IDO1 | TNFRSF4 | KIR2DL3 | BTLA | TNFRSF14 |
| ADORA2A | CTLA4 | TNFRSF18 | IL2RA | HAVCR2 | PDCD1 |
| TIGIT | TNFRSF9 | ICOS | TLR4 | CD27 | LAG3 |
| CD40 | KIR2DL1 | EDNRB |  |  |  |

**Table S10. The summary of data utilized in this study.**

| **Rank** | **Data** | **Source** |
| --- | --- | --- |
| 1 | Pan-cancer TCGA | UCSC Xena  (http://xena.ucsc.edu/public/) |
| 2 | GTEx | UCSC Xena  (http://xena.ucsc.edu/public/) |
| 3 | 38 scRNA datasets for 19 cancer types | TISHC2  (http://tisch.comp-genomics.org) |
| 4 | 6 external validation datasets for prognostic model | GEO  (https://www.ncbi.nlm.nih.gov/geo)  GSE15459, GSE130219, GSE42568, GSE50081, GSE63885, GSE72094 |
| 5 | 9 immunotherapy bulk-level cohorts | Details were summarized in the corresponding tables |
| 6 | 1 immunotherapy single cell-level cohort | PMID 31359002 |
| 7 | 75 immune checkpoint genes | PMID 32814346 |
| 8 | signatures of HALLMARK and C6 genesets | MsigDB  (https://www.gsea-msigdb.org/gsea/msigdb) |
| 9 | 10 hypoxia-related  gene signatures | Details were summarized in the corresponding tables |
| 10 | 17 Immune Screening  Datasets | Details were summarized in the corresponding tables |
| 11 | 1078 cell lines with  CERES scores | DepMap  (https://depmap.org/portal/download/all/) |
| 12 | 34 datasets for validating the performance of *HYP.SIG* | Details were summarized in the corresponding tables |
